# *Kluyveromyces lactis* and *Saccharomyces cerevisiae* for Fermentation of Four Different Coffee Varieties

**DOI:** 10.3390/foods14010111

**Published:** 2025-01-03

**Authors:** Danilo José Machado de Abreu, Denis Henrique Silva Nadaleti, Rafaela Pereira Andrade, Tamara Leite dos Santos, Dérica Gonçalves Tavares, Cesar Elias Botelho, Mário Lúcio Vilela de Resende, Whasley Ferreira Duarte

**Affiliations:** 1 Instituto Nacional de Ciência e Tecnologia do Café (INCT), Lavras CEP 37203-202, MG, Brazil; danilo.mabreu@gmail.com (D.J.M.d.A.); rafaelaandrade1210@gmail.com (R.P.A.); tamaraleitesantos@gmail.com (T.L.d.S.);; 2 Biology Department, Federal University of Lavras (UFLA), Lavras CEP 37203-202, MG, Brazil; 3 Empresa de Pesquisa Agropecuária de Minas Gerais (EPAMIG), Lavras CEP 37203-202, MG, Brazil; denis.nadaleti@epamig.br (D.H.S.N.); cesarbotelho@epamig.br (C.E.B.); 4 Department of Biology, University of Louisville, Louisville, KY 40208, USA; derica.goncalvestavares@louisville.edu

**Keywords:** coffee fermentation, arabica coffea, bioactive compounds, coffee quality

## Abstract

One strategy for adding unique characteristics and flavors to improve coffee quality is the selection of starter microorganisms. Here, we aimed to evaluate the effect of *Saccharomyces cerevisiae* LNFCA11 and *Kluyveromyces lactis* B10 as starter cultures on the quality of four different wet-fermented coffee varieties. Microbiological, molecular, and chemical analyses were carried out to identify yeast, bacteria, volatile compounds, carbohydrates and bioactive compounds in coffee. Sensory analysis was performed by Q-graders certified in coffee. Starter yeasts affected bioactive and volatile compounds as well as sensory descriptors in the coffee varieties. *S. cerevisiae* CA11 allowed a higher content of trigonelline and chlorogenic acid in MGS Paraíso 2 (P2) and Catuai Amarelo IAC62 (CA62) varieties. *K. lactis* B10 fermentation resulted in higher chlorogenic acid only on the P2 cultivar and MGS Catucaí Pioneira (CP). In addition, 5-methyl-2-furfuryl alcohol and n-hexadecanoic acid were produced exclusively by yeast fermentation compared to spontaneous fermentation. The coffee cultivars P2 presented more complex sensory descriptors and the attributes of aroma, acidity, and balance when fermented with *S. cerevisiae* CA11. Sensory descriptors such as lemongrass, citrus, and lemon with honey were related to *K. lactis* B10. Starter cultures allowed the coffees to be classified as specialty coffees. The fermentation showed that the choice of starter yeast depends on the desired sensory descriptors in the final product.

## 1. Introduction

Although coffee is not a basic nutritional crop, it has become highly economically significant globally. In 2021, coffee production exceeded 150 million bags, while global consumption reached 173.1 million. Brazil has a favorable climate for this crop and is one of the world’s largest coffee producers and exporters, producing approximately 69 million bags in 2023 [1]. Additionally, Arabica coffee (*Coffea arabica*) accounts for approximately 70% of the national production. By 2024, the estimated increase in production will be 4.7% over that of the 2023 crop [2,3,4].

Due to price increases, coffee beverages are nowadays considered a consumer experience and not just a valuable crop previously characterized for a mass consumption [4,5]. With the introduction of this concept, specialty coffees, defined as beverages of exceptional quality distinguished by their unique flavors and characteristics, have been increasing in popularity. This is mainly due to the change in consumer behavior, which values the pleasure of consumption, health, and sustainability [6].

This market niche was only possible due to the promising cultivation of coffee varieties, good management of post-harvest processing methods, and the use of starter cultures for fermentation. These are factors that may influence the sensory quality of the beverage. Starter cultures accelerate the fermentation process [7], directly influencing the biochemical composition and the volatile compounds in coffee and contributing to obtaining beverages with distinct and desirable sensory profiles. Several studies have shown that coffee quality improves with the use of *Pichia fermentans*, *Pichia guilliermondii*, *Saccharomyces cerevisiae*, *Torulaspora delbrueckii*, and *Kluyveromyces fragilis* [8,9,10]. It has been reported in the literature that the behavior of microorganisms, especially yeasts, varies according to the genetic diversity among coffee varieties, production regions, altitudes, processing methods, and microbial species [11,12,13,14].

Selecting specific microorganisms for the fermentation process is essential to understand their role and how they can improve coffee quality. In the case of yeasts, they can give coffee a higher acidity than naturally fermented coffees. In addition, authors have shown that sensory descriptors differed in the presence of starter yeasts. *Toluraspora delbrueckii*, for example, allowed the perception of citrus fruits in the Bourbon Amarelo and Canário Amarelo varieties [15]. The same yeast was also used to ferment other arabica coffee varieties, including Catuaí Vermelho IAC-44, which made it possible to imprint other sensory descriptors related to chocolate and cucumber when used in the post-harvest processing of low-altitude coffees [9]. The fermentation of *Coffea canephora* in the presence of *Meyerozyma caribbica* not only reduces the presence of filamentous fungi during fermentation but also raises the coffee score by 2 points when the population is above 5 log CFU.g^−1^ at the end of 72 h of fermentation [16].

Most studies using yeasts for coffee fermentation have focused on *S. cerevisiae* and *T. delbrueckii*. Although *K. lactis* has been isolated from coffee fermentation, it has not yet been reported as a starter culture for coffee. In other fermented products such as cheese and wine, the relevant role of this yeast in the production of various aromatic compounds is recognized, mainly in the group of esters whose most frequent aroma descriptors are “floral and fruity”. In addition to the impact on the composition of desirable volatiles, *K. lactis* may also impact the composition of bioactive compounds, as already described in the literature for coffee fermentation with other yeasts. In recent years, our research group has been studying the potential of *K. lactis* because of its enzymatic, metabolic, technological, and functional activities. Therefore, the objective of this study was to evaluate *K. lactis* B10 as starter culture for wet fermentation of four different Arabica coffee varieties compared to *S. cerevisiae* LNF CA11.

## 2. Materials and Methods

### 2.1. Coffee Beans

Four varieties of Arabica coffee (*C. arabica*) were evaluated: Catuaí Amarelo IAC 62 (CA62), MGS Paraíso 2 (P2), MGS Ametista (A), and MGS Catuaí Pioneira (CP). The coffees were harvested in a farm in Lavras, Minas Gerais, Brazil (21°17′14.0″ S 45°05′59.8″ W, 870 m). The washing was performed in a polyethylene box with a capacity of 500 L to separate and remove fruits with lower density and impurities. After this washing, 8 kg of fruit were collected at the ripe stage and peeled. This process was performed for all the varieties separately [9].

### 2.2. Inoculation and Fermentation

The yeasts used in this study were *S. cerevisiae* LNF CA11 (LNF-Latin America, Bento Gonçalves, Brazil) and *K. lactis* B10 [17] (previously stored in 20% glycerol (*v*/*v*) at −80 °C). The yeasts were reactivated in 1 mL of YPD (1% yeast extract, 2% peptone, 2% dextrose) at 30 °C for 24 h. Cell growth was triggered by diluting cultures at a ratio of 1:10 in increasing volumes until the desired number of cells was obtained.

The harvested and peeled coffee fruits were processed using wet fermentation for 48 h [9]. The pulped coffee beans (3.5 kg of coffee beans for each treatment) were distributed in closed polypropylene fermenters with 9 L of water. The room temperature ranged from 20–24 °C, and the beans were shaken every 24 h. For each variety, three different fermentations were performed: spontaneous fermentation (N), inoculation with *S. cerevisiae* LNF CA11 (CA11), and inoculation with *K. lactis* B10 (B10). All fermentations were performed in triplicate. After fermentation, the coffee beans were laid out to dry in the sun on suspended terraces in a thin layer (7 L/m^2^). The layer of coffee beans was turned according to the moisture loss to allow slow and uniform drying until the water content reached 10.8–11.2% [18]. After drying, the samples were packed in waterproof plastic bags and stored in a cold room (at around 16 °C) for 40 days to ensure that the water content in the grains was uniform. After this period, the samples were processed and standardized for chemical and sensory analysis.

### 2.3. Microbiological Analyses

#### 2.3.1. Microbial Isolation and Counting

Aliquots were collected at 0, 24, and 48 h of fermentation to evaluate the microbiota present during fermentation. Ten grams of each treatment was added to a 0.01% (*w*/*v*) peptone water solution and shaken on an orbital shaker at 200 rpm for 30 min. Mesophilic bacteria were cultured on PCA culture media (5 g·L^−1^ tryptone, 1 g·L^−1^ glucose, 2.5 g·L^−1^ yeast extract, and 15 g·L^−1^ agar); Man, Rogosa, and Sharpe agar (MRS) was used for lactic acid bacteria (LAB). Both PCA and MRS were supplemented with 0.2% nystatin (*v*/*v*). YPD (yeast extract: 10 g·L^−1^; peptone: 20 g·L^−1^; dextrose: 20 g·L^−1^; agar 15 g·L^−1^ distilled water) supplemented with 0.01% (*w*/*v*) chloramphenicol was used for yeast isolation. Bacteria were incubated in media at 37 °C for 48 h, while yeasts were incubated in YPD media at 28 °C for 48 h. The microorganism counts are expressed in colony-forming units per g of coffee (log CFU.g^−1^). Serial dilutions were carried out to ensure better representation of the microorganisms present. Yeasts and bacteria were isolated, and their morphotype and predominance during fermentation were characterized. Thus, they were purified on YPD, MRS, and PCA agar and stored in 20% glycerol (*v*/*v*) at −80 °C.

#### 2.3.2. DNA Extraction, Amplification, and Sequencing

For DNA extraction, the yeasts were reactivated by inoculating 50 µL of the cultures in 1 mL of YPD medium, incubated at 28 °C for 24 h, incubated with another 4 mL of YPD, and incubated at 28 °C for another 24 h. The bacteria were reactivated by inoculating 100 µL of the cultures into 5 mL of MRS and PCA medium and incubated at 37 °C for 24 h. Then, 100 µL of the reactivated cultures were inoculated into 5 mL of MRS and PCA and incubated at 37 °C for another 24 h. The cultures were centrifuged, and the DNA was extracted from yeast and bacteria with the Promega Wizard^®^ DNA Extraction Kit (Madison, WI, USA) following the manufacturer’s instructions. For the molecular identification of yeasts, the 5.8S region of the internal transcription spacer (ITS) was amplified using the primers ITS1 (5′-TCCGTAGGTGAACCTGCGG-3′) and ITS4 (5′-TCCTCCGCTTATTGATATGC-3′) [19]. For the identification of bacteria, the 16S1 (5′-CGCTAGTAATCGCAGGTCA-3′) and 1492R (5′-CGGCTACCTTGTTACGACTT-3′) primers were used to amplify the V1 and V9 regions of 16S rRNA [20]. A Hot Start Taq 2X Master Mix (NEB) kit (Ipswich, MA, USA) was used for polymerase chain reaction (PCR) according to the manufacturer’s recommendations. The PCR conditions for the 16S region were 95 °C for 1 min, 95 °C for 15 s, 55 °C for 30 s, 72 °C for 30 s and 72 °C for 2 min for 35 cycles. For amplification of the internal transcription spacer (ITS), 35 cycles of 95 °C for 1 min, 95 °C for 15 s, 51 °C for 30 s, 72 °C for 30 s, and 72 °C for 2 min were performed. Subsequently, the PCR reaction was verified on 0.8% agarose gel, and the samples were stored at −20 °C.

#### 2.3.3. Phylogenetic Analysis

Consensus sequences were assembled from forward and reverse sequences using SeqAssem ver. 07/2008 (SequentiX, Digital DNA Processing, Klein Raden, Germany). Additional sequences were obtained from the GenBank database. Phylogenetic analyses were performed using MEGA X (version 10.0). The sequences were aligned using CLUSTAL W. Maxima likelihood (ML) and neighbor-joining (NJ) phylogenetic trees were constructed for each gene. The most appropriate replacement model, determined based on the lowest Akaike information criterion, was built using MEGA X. Model T92+G was selected for ITS, and K2+I was selected for 16S. ML-based analyses were performed by calculating an initial tree using BioNJ; the subsequent heuristic search was performed with the nearest-neighbor-interchange (NNI) option. Clade support was inferred from 1000 bootstrap replications [21].

### 2.4. Chemical Analyses

#### 2.4.1. Carbohydrates

Glucose and fructose were analyzed during fermentation (0, 24, and 48 h), and extraction was performed according to Murkovic and Derler [22] with some modifications. First, the samples were ground with liquid nitrogen in an IKA A11 mill until a fine powder was obtained. Then, 0.5 g of each sample was diluted in 3.4 mL of Milli-Q water and placed in an ultrasonic bath for 10 min. After this step, each sample was vortexed, centrifuged at 9000 rpm/10 min/4 °C, and filtered through 0.22 µm membranes.

Chromatographic analyses were performed using a Shimadzu UFLC (Kyoto, Japan) equipped with an LC-20AT high-pressure quaternary pump, a DGU-20A5 degasser, a CBM-20A interface, and a SIL-20A-HT automatic injector. The detection was performed using the refractive index (RID 20A), and separation was performed using a Supelcogel 8H column (Supelco, Bellefonte, PA, USA). The mobile phase for the elution of the compounds was a solution of 5 mM sulfuric acid in isocratic mode at 30 °C in an oven, and the injection volume was 20 µL. Each compound was identified by comparing the retention times of the peaks of the experimental sample with the retention times of reference standards injected under the same conditions. Quantification was performed using external calibration [17,23].

#### 2.4.2. Bioactive Compounds

The bioactive compounds analyzed were trigonelline, chlorogenic acid (5-caffeoylquinic acid), and caffeine. For the analysis of these bioactive compounds, the coffee beans were collected during fermentation (0, 24, and 48 h). The raw coffee beans were ground in a ball mill with liquid nitrogen. To extract bioactive compounds, 0.5 g of ground sample was diluted in 50 mL of distilled water at the boiling point and kept for 3 min in a water bath. The samples were filtered through 0.22 µm membranes and then analyzed.

Chromatographic analyses were performed on the same HPLC mentioned above using a UV–Vis detector (SPD-20A). The column used was a Supelcosil LC-C18 column (4.6 × 250 mm, 5 µm) connected to a Supelcosil C18 precolumn (4.6 × 12.5 mm, 5 µm). The mobile phase used was a solution of 1% acetic acid (Solvent A) and methanol:water: acetic acid (85:14:1% *v*/*v*) (Solvent B). The samples were eluted isocratically. The wavelength was 272 nm, the flow rate was 1.0 mL min^−1^, and the injection volume was 20 µL. Each compound was identified by comparing the retention times of the peaks of the experimental sample with the retention times of reference standards injected under the same conditions. Quantification was performed by external calibration [24].

#### 2.4.3. Validation Parameters of the HPLC Methods

To ensure the reliability of analyses, the linearity, limit of detection (LOD), limit of quantification (LOQ), precision, and accuracy of HPLC methods were determined [24,25,26].

Linearity was expressed through the equation of the line and the respective coefficient of determination (R2). Parameters related to the constructed analytical curve were considered to determine the LODs and LOQs. The following mathematical relationships were used to determine the limits: LOD = 3 × (s/S) and LOQ = 10 × (s/S), where s is the estimate of the standard deviation of the regression line equation, and S is the slope of the analytical curve. Precision was assessed using intermediate precision. The precision of the method was estimated by the coefficient of variation (CV) of a series of measurements using the following mathematical equation: CV (%) = (s/CMD) × 100, where s is the estimate of the standard deviation, and DMC is the mean concentration determined. The accuracy was evaluated by means of recovery tests using three randomly chosen samples, which were fortified with analyte standards at three different concentrations (low, medium, and high). Recovery was determined considering the results obtained for each analyte studied using the following mathematical equation: %Recovery = [(measured concentration)/(expected concentration)] × 100.

#### 2.4.4. Volatile Compounds

The analysis of volatile organic compounds was performed as described by Pereira et al. [27] with some modifications. The analyses were performed on fermented, dry, and roasted coffee (48 h). One gram of each sample was added to 15 mL flasks and extracted using HS–SPME with a 50–30 µm DVB/CAR/PDMS fiber (Supelco) for 30 min at 70 °C. The fiber was kept in the injector for 5 min for the desorption of volatiles, and the analysis was performed in a gas chromatograph coupled to a GC–MS mass spectrometer (QP2010E, Shimadzu Corp. Kyoto, Japan) equipped with an RTX-5MS column (30 m × 0.25 mm). Helium was used as the carrier gas with a 1.67 mL/min flow rate. The inlet temperature was 250 °C, the detector temperature was 300 °C, and the injections were performed in splitless mode. The initial column temperature was 40 °C and maintained for 5 min, after which it was increased by 3 °C/min until reaching 180 °C and then increased by 10 °C/min again until reaching 250 °C and maintained for 5 min. The compounds were identified using the NIST library version 2011 and confirmed based on the linear retention index calculated using a homologous series of alkanes. The concentrations are expressed as equivalents of 4-methyl -2-pentanol. The relative percentage of each compound was calculated by normalizing the peak area [28].

### 2.5. Sensory Analysis

The coffee samples, standardized to a sieve size of 16 and above, free of intrinsic and extrinsic defects, were roasted according to the protocol proposed by the Specialty Coffee Association (SCA). The roasting resulted in a coloration ranging from 55# to 65# on the Agtron scale for whole beans, with roasting times between 8 and 12 min [29].

Sensory analyses were performed on five cups per sample by three calibrated Q-graders, following the same protocol. This protocol consists of ten sensory attributes, namely, fragrance/aroma, flavor, finish, acidity, body, balance, and general, which were evaluated with scores in the range of 6 to 10 points each. The attributes of uniformity, sweetness, and clean cup were also evaluated, and 2 points were assigned per cup (for coffees free of defects, uniform, and with a minimum sweetness equivalent to a concentration of 0.5% *m*/*v* sucrose). The total sensory score was calculated considering the sum of the ten sensory attributes mentioned, with coffees receiving a total score of 80 points or higher being classified as specialty coffees. In addition, the judges noted all the perceptible nuances in the sensory attributes of the samples.

### 2.6. Statistical Analysis

A factorial completely randomized design (CRD) was used, in which the factors were as follows: 4 varieties of arabica coffee (Catuaí Amarelo IAC 62, MGS Paraíso 2; MGS Ametista and MGS Catuaí Pioneira) and 3 types of inoculation (spontaneous fermentation, inoculation with *S. cerevisiae* LNF CA11 and inoculation with *K. lactis* B10). The results are expressed as the mean ± standard deviation, to which analysis of variance (ANOVA) was applied, followed by the Scott–Knott test (*p* < 0.05) for the bioactive compounds, carbohydrates, and sensory analysis. Multivariate statistical methods, principal component analysis (PCA), and hierarchical cluster analysis (HCA) were applied to compare the results obtained during coffee fermentation. A heatmap was constructed to evaluate the volatile compounds profiles. Pearson correlations were determined to compare the obtained results from fermentation. All the statistical analyses were performed using R software (4.0.2, FactoMineR, and factoextra packages). A Venn diagram was constructed to evaluate the volatile compounds of roasted coffee using a free online program (https://web.rniapps.net/netsets/ (accessed on 20 May 2024)).

## 3. Results and Discussion

### 3.1. Microbial Populations

**Figure 1:**
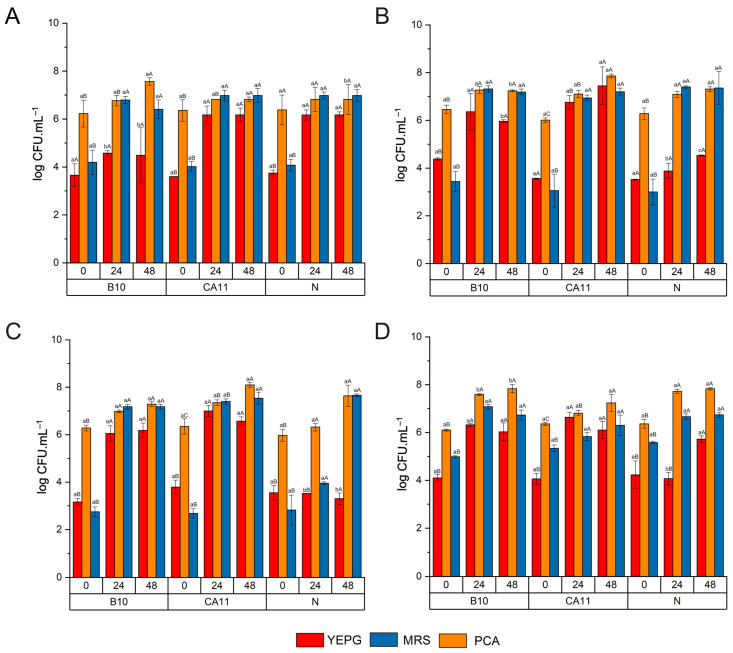
Variation in the microbial population (log CFU.mL^-1^) of coffee cultivars during fermentation. The fermentation time is 48 h. (**A**) CA62; (**B**) A; (**C**) P2; (**D**) CP. (N: Natural fermentation; CA11: Fermentation inoculated with *S. cerevisiae*. LNF CA11; B10: Fermentation inoculated with *Kluyveromyces lactis* B10.) Different lowercase letters indicate significant differences according to the Scott–Knott test (*p* < 0.05) when comparing the interaction between coffee variety and fermentation time. Different capital letters indicate a significant difference according to the Scott–Knott test (*p* < 0.05) when comparing the interaction between coffee variety and inoculation.

The microbial groups monitored varied throughout fermentation. Therefore, there was a significant difference between the monitored populations, depending on the microbial group evaluated and the coffee variety (Figure 1).

Yeasts and filamentous fungi were counted in YPD. At the beginning of the fermentation, these organisms varied by approximately 4 log CFU.mL^−1^. The final yeast population under conditions of natural fermentation depended on the fermented coffee variety (Figure 1). Coffee varieties CP and CA62 presented an average yeast population at the end of fermentation of 5.94 log CFU.mL^−1^, while varieties A and P2 had an average yeast population of 3.9 log CFU.mL^−1^. There was a significant difference in the yeast population when observing the fermentation of coffee varieties inoculated with yeast starter cultures (*p* < 0.05). In general, there was an increase in the yeast population during fermentation. The yeast populations of varieties A, CA62, and CP inoculated with *S. cerevisiae* CA11 were 109.26%, 78.33%, and 50.24% greater, respectively, than those inoculated with *K. lactis* B10 after 48 h of fermentation. Therefore, yeast growth was affected by the coffee variety used.

The population behavior of other evaluated microbial groups during fermentation also depended on the coffee variety. The most significant variation in LAB occurred in the P2 variety, followed by the A, CA62, and CP varieties. Similarly, significant variation in the mesophilic aerobics measured in the PCA medium was also noted (Figure 1). During coffee fermentation, aerobic bacteria have a larger microbial population than LAB, a behavior favored by the employed wet processing method [12].

This population behavior is variable due to the substrate concentration available at the beginning of fermentation, which was different for each coffee variety. Coffee fermentation is complex and depends on a succession of microbial populations [9]. Given that the bacterial population was greater than that of yeasts in all the treatments evaluated, the permanence and competition of the starter cultures during fermentation are essential because their metabolic activity may be related to the differences in the chemical compounds evaluated in the coffee and possibly to the correlated sensory characteristics [30].

### 3.2. Identification of Microorganisms, Phylogeny, and Predominance

**Figure 2:**
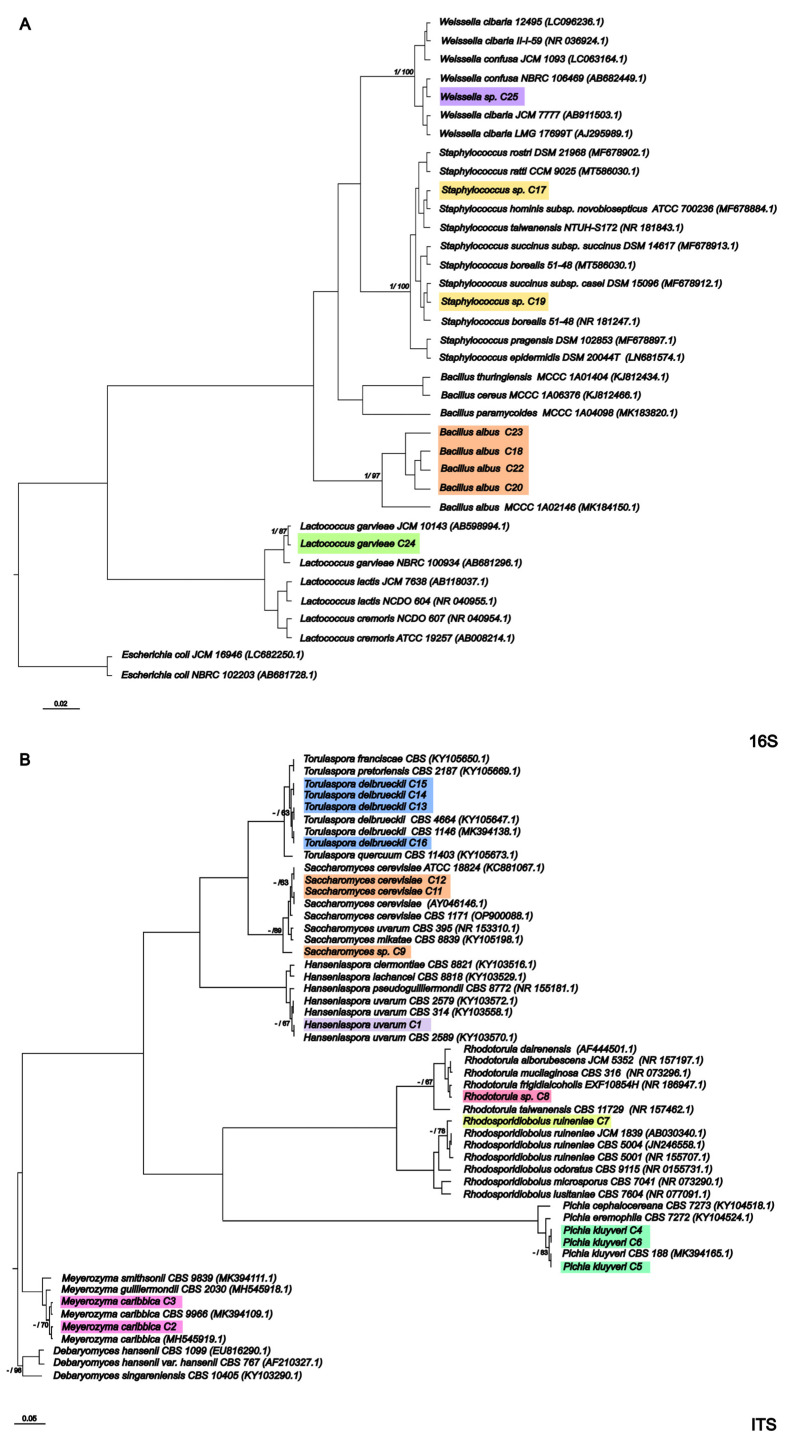
Phylogenetic tree showing the relationships between the identified isolates and microorganisms. (**A**) Sequences of the 16S rRNA gene. The 16S rRNA gene from the *Escherichia coli* strains was selected as the outgroup. (**B**) Sequences of the ITS rRNA gene. The ITS rRNA genes of strains of the genus *Debaryomyces* sp. were selected as the outgroup. The tree was obtained using the neighbor joining (NJ)/maximum likelihood (ML) method of the Mega software package version 10.0 with a bootstrap value of n = 1000.

Sixteen yeast and eight bacteria were identified during the fermentation of coffee varieties. The phylogenetic trees obtained using Bayesian and ML analysis contained the same clades (Figure 2). Phylogenetic analysis of the 16S rRNA gene revealed that isolates C23, C22, C20, and C18 formed a monophyletic group with high posterior Bayesian support (1) and ML bootstrap (97), closely related to species *Bacillus albus*. On the other hand, isolate C24 clustered with a species of *Lactococcus garvieae*, also with high posterior Bayesian support (1) and ML bootstrap (Figure 2A). The phylogenetic analysis of the 16 rRNA gene grouped isolates C17 and C19 with species of the genus *Staphylococcus* (posterior/bootstrap = 1/100) and isolate C25 with species of the genus *Weissela* (posterior/bootstrap = 1/100). The 16S rRNA gene could not discriminate between *Staphylococcus* sp. and *Weissela* species because only the V1 and V9 regions were used.

Phylogenetic analyses of the ITS rRNA gene grouped isolate C9 together with species of the genus *Saccharomyces* (posterior/bootstrap = -/89) and isolate C8 with species of the genus *Rhodutorula* (posterior/bootstrap = 1/67) (Figure 2B). These isolates were only identified at the genus level as *Saccharomyces* sp. and *Rhodutorula* sp. Despite this, the gene was sufficient to identify isolates C16, C15, C14, and C13 at the species level as *Torulaspora delbrueckii* (posterior/bootstrap = -/63); isolates C11 and C12 as *S. cerevisiae* (posterior/bootstrap = -/63); isolates C5, C6, and C4 as *Pichia kluyveri* (posterior/bootstrap = -/83); isolate C1 as *Hanseniaspora uvarum* (posterior/bootstrap = -/83); isolate C7 as *Rhodosporidloobolus ruineiae* (posterior/bootstrap = -/78); and isolates C2 and C3 as *Meyerozyma caribbica* (posterior/bootstrap = -/70).

**Figure 3:**
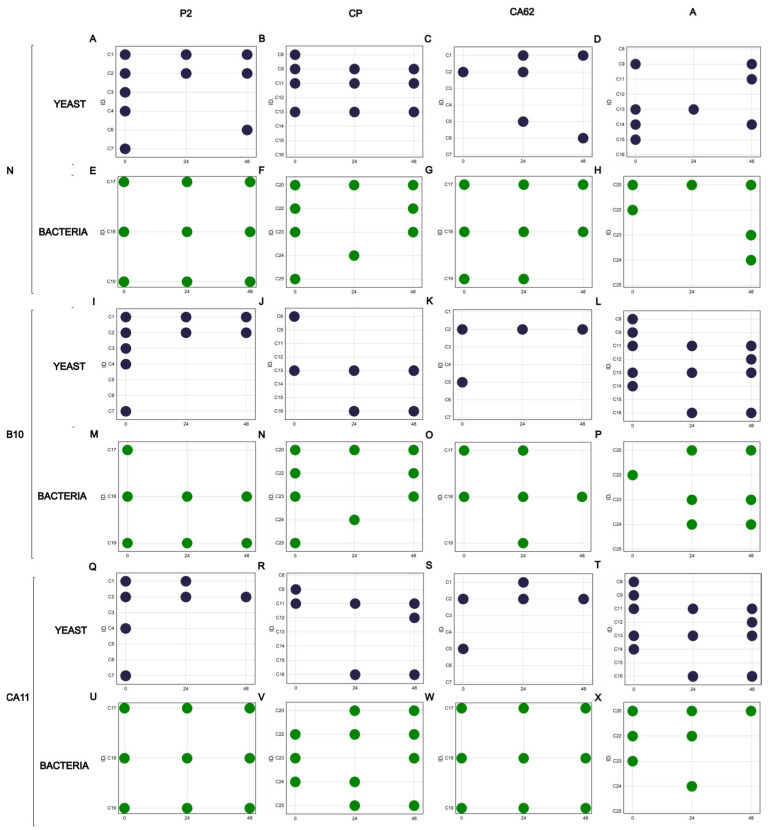
Bubble plot showing the absence of microorganisms identified using the 16S rRNA and ITS genes during fermentation of different arabica coffee cultivars. ID: identification of microorganisms. The presence of yeast is represented in blue; the presence of bacteria is represented in green. (Microorganism present in N: (**A**–**H**); B10: (**I**–**P**); CA11: (**Q**–**X**)).

Given the identification of the microorganisms, it was possible to construct a bubble chart to show the presence and absence of the microorganisms throughout the fermentations varies according to the coffee variety analyzed and the type of starter culture used (Figure 3).

Sixteen yeasts were isolated during the fermentation process. Analyzing their presence, it was observed that the identified species *M. caribbica* C2 was the only yeast that was present in all established fermentation processes. Considering that the inoculation of starter cultures modulated the presence of the species, *Hanseniospora uvarum* C1 was present only in the fermentation of the P2 variety when *K. lactis* B10 was used as the starter culture. The same behavior was observed for *P. kluyveri* C5, identified only in the CP variety and in the yeast inoculated treatments. Despite the phylogenetic proximity of the identified *P. kluyveri* strains, the C4 and C6 strains were present only at the beginning and end of fermentation, respectively, and in the coffee varieties CA62 and P2.

Regarding the presence of the genus *Torulaspora*, strain C13 was present throughout the fermentation process of treatments N and *K. lactis* B10 and only in varieties A and CP, while strains C16, C14, and C15 were only present at 24 and 48 h and the beginning of fermentation, respectively. *Rhodosporidiobolus ruminae* strain C8 was identified only during the initial fermentation of the CP and A varieties, along with microorganisms of the genus *Rhodotorula*. These yeasts have already been reported to be involved in wet coffee fermentation and may improve sensory properties by producing volatile compounds and inhibiting the growth of ochratoxigenic filamentous fungi [10,11,14] (Figure 3).

Analysis of the presence of bacteria revealed that *Bacillus* (C18, C20, C22, and C23), *Staphylococcus* (C19 and C17), and *Lactococcus* (C24) were present in the fermentation process of the coffee varieties. On the other hand, *Weissela* sp. (C25) was present only in the inoculated treatments and in the CP variety. In the *S. cerevisiae* CA11 inoculation, it appeared only at 24 and 48 h. The opposite behavior occurred for the inoculation with *K. lactis* B10, where it appeared only at the beginning of fermentation. This behavior suggested that inoculation may have favored the growth of this bacterium in the presence of yeasts. In addition, the presence of this genus in coffee fermentation has already been reported in the literature; in addition to contributing to the quality of the fermentation process through the production of lactic acid, it also contributes to the inhibition of the growth of filamentous fungi [11,14].

### 3.3. Evaluation of the Effect of Starter Cultures on Bioactive Compounds and Carbohydrates

**Figure 4:**
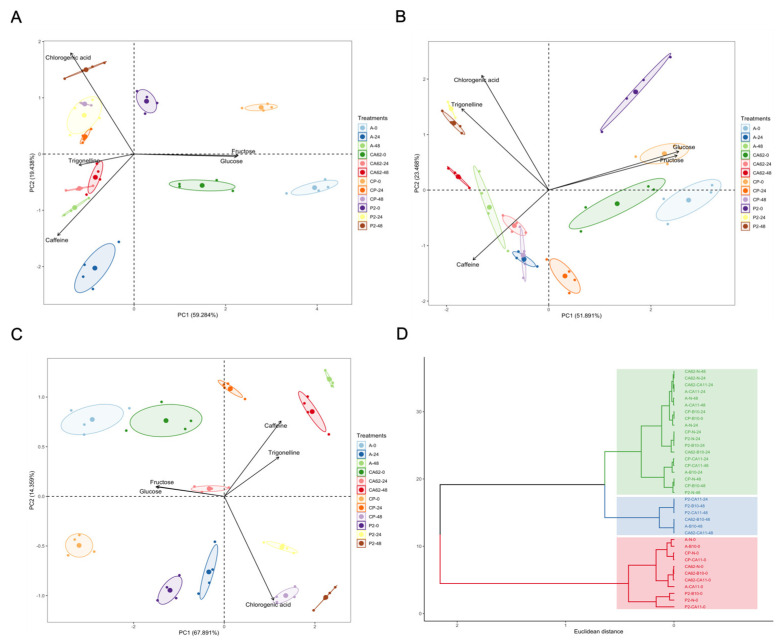
Multivariate analysis of fermented coffee cultivars. Principal component analysis (PCA) (**A**): N; (**B**): CA11; (**C**): B10; (**D**): Hierarchical cluster analysis (HCA).

The contents of carbohydrates and bioactive compounds were analyzed throughout fermentation. Monitoring the coffee fermentation process is essential to ensure that the full fruit quality potential is developed and remains in the coffee beans. One of the parameters to be monitored during the fermentation process is the consumption of carbohydrates, as they indicate the population growth of microorganisms and the production of volatile and nonvolatile metabolites. Principal component analysis (PCA) was performed to intuitively evaluate the data presented in Table S2 (Supplementary Material). The use of starter cultures was related to the accumulation of bioactive compounds, depending on the coffee variety. In addition, treatments were clearly separated according to fermentation time. According to these results, the variables evaluated in this study, including coffee processing methods, the type and altitude of the roasting conditions, and the variety, preparation, and origin of the coffee, all affect the content of bioactive compounds (Figure 4) [9,14,31].

At the beginning of fermentation, high concentrations of glucose and fructose were detected in all coffee varieties analyzed and in the treatments using starter cultures. According to Pearson’s correlation, the bacteria-bacteria-carbohydrate and bacteria-yeast-carbohydrate ratios were strong and significant in the fermentation of all the varieties and were fundamental for allowing the substrate to be consumed. As a result, all fermentations showed carbohydrate consumption after 48 h of fermentation (Supplementary Material—Figure S3). PCA of the spontaneous fermentation of the coffee varieties explained 78.72% of the variance, whereas the components PC1 and PC2 represented 59.28% and 19.43%, respectively (Figure 4A). On the positive side of PC1, all four coffee varieties were correlated with glucose and fructose at the initial fermentation time (Figure 4A). On the other hand, after 48 h of fermentation, variety P2 was characterized by higher chlorogenic acid content, CA62 correlated to trigonelline, and variety A was characterized by caffeine content (Figure 4A).

Regarding fermentation with *S. cerevisiae* CA11, 75.35% of the data variance was explained by PC1 (51.89%) and PC2 (23.46%) (Figure 4B). In this sense, glucose and fructose influenced the separation of PC1, and chlorogenic acid and trigonelline influenced the separation of PC2 (Supplementary Material–Figure S2B). Bioactive compounds such as caffeine accumulated mainly in coffee varieties A, CP, and CA62, while P2 has a higher content of chlorogenic acid and trigonelline. Inoculation of *S. cerevisiae* CA11 resulted in faster glucose consumption in the first 24 h of fermentation. This tendency was observed in the fermentation of all the varieties. However, for variety A, this consumption was more significant at 24 h of fermentation, with a reduction of 86.51% in the glucose content (T0: 0.89 g·L^−1^; T48: 0.12 g·L^−1^) (Figure 4B). Given this behavior, the presence of *Lactococcus garvieae* and *Bacillus albus* contributed to increased glucose consumption in coffee variety A, while the presence of *Weisella* sp. seems to have influenced the concentration of glucose and fructose only in the CP variety, where glucose consumption was 91.02% (T0: 0.78 g·L^−1^; T48: 0.07 g·L^−1^) and fructose consumption was 88.62% (T0: 1.67 g·L^−1^; T48: 0.19 g·L^−1^) (Figure 3X,V). In the other varieties, namely, CA62 and P2, *Bacillus albus* C18 may have helped consume the sugars since they were present throughout the fermentation process (Figure 3W,U). Thus, LAB and mesophilic bacteria showed strong correlations (above 0.92) with sugar consumption.

Regarding the fermentation PCA of the varieties in the presence of *K. lactis* B10, 82.25% of the data was explained (Figure 4C). Glucose and fructose influenced the separation of PC1, and caffeine and chlorogenic acid influenced the separation of PC2 (Supplementary Material—Figure S2C). Unlike the coffees inoculated with *S. cerevisiae* CA11, a different bioactive compound accumulation profile was observed. The Ametista variety and CA62 accumulated trigonelline and caffeine at the end of fermentation. However, at 24 h, these varieties had the same concentrations of these bioactive compounds as noted at the start of fermentation. The metabolism of the starter culture may have influenced this. The CP and P2 varieties accumulated the highest concentrations of chlorogenic acid.

The sugar consumption for *K. lactis B10* differed from that observed for *S. cerevisiae* CA11. After 24 h of fermentation, only 55.22% of glucose and fructose were consumed for coffee variety A (glucose: 0.30 g·L^−1^; fructose: 0.65 g·L^−1^) and 70% (glucose: 0.24 g·L^−1^; fructose: 0.52 g·L^−1^) for CP variety (Figure 4C). At the end of fermentation (48 h), 85% of carbohydrates were consumed for both varieties. There is a difference between *S. cerevisiae* and *K. lactis* in regulating mitochondrial respiration [32]. Although both are considered yeasts in the facultative aerobic group, *K. lactis* is an aerobic respiratory yeast, not a fermentative one. Therefore, its tendency to consume sugars differs due to its preference for non-fermentable oxidative and oxidative-reductive pathways [33]. Concerning sugar consumption, the different microbial profiles for each studied coffee variety probably impacted the consumption of these sugars. The permanence and competition of starter cultures during fermentation are essential because their metabolic activity may be related to the differences in the chemical compounds evaluated in the coffee and possibly to the correlated sensory characteristics [30].

In general, a strong correlation (>−0.66) between the yeasts and the sugars, together with this starter culture, also helped the sugar consumption process (Supplementary Material—Figure S3). This is mainly because the yeasts enabled the symbiotic development of the LAB through cell autolysis. On the other hand, LAB can help in the fermentation process, mainly because they are involved in producing organic acids and consuming carbohydrates. This relationship impacts the flavor and aroma profile [12,34].

Among the analyzed bioactive compounds, chlorogenic acid presented the highest content in all coffee varieties, followed by caffeine and trigonelline. Trigonelline, a bioactive compound, contributes mainly to the roasted coffee flavor because it gives rise to volatile compounds in the pyrrole and pyridine chemical classes. Caffeine confers bitterness to the beverage, and chlorogenic acid is responsible for pigmentation, astringency, and the production of volatile phenols since part of this compound is degraded when the beans are roasted [35,36]. Considering their properties related to sensory characteristics, the accumulation of bioactive compounds in the presence of starter cultures will influence the flavor of the final coffee beverage.

A strong and significant correlation with bioactive compounds was observed with increasing microbial population concentration, regardless of the starter culture and coffee variety. The retention of chlorogenic acid in coffee variety CA62 (0.74) may have been supported by mesophilic bacteria quantified using PCA agar. This group of microorganisms helps with the availability of chlorogenic acid by modulating precursors or intermediates related to this compound, favoring its retention or transformation into desirable bioactive compounds. In addition, in varieties A (MRS: 0.73; YPD: 0.83; PCA: 0.74) and P2 (MRS: 0.86; YPD: 0.78; PCA: 0.79), it was also possible to observe strong and significant correlations between all the microbial groups evaluated and chlorogenic acid.

Although the mechanism of production and transformation of bioactive compounds by coffee-related microorganisms has not been fully elucidated, yeasts such as *Hanseniaspora uvarum*, *Pichia kluyveri*, and *Saccharomyces cerevisiae*, as well as the bacteria *Bacillus albus* and *Lactococcus garvieae* identified in this study have enzymatic capacity through pectinolytic enzymes and esterases, which contribute to the degradation of mucilage and soluble fibers and the biotransformation of compounds such as chlorogenic acid during the wet fermentation of coffee beans. Microorganisms such as LAB have an enzymatic mechanism rich in transferases and esterases. However, they do not have demethylases. This enzyme has the function of attaching a methyl group to the hydroxyl group they have. Chlorogenic acids, however, are synthesized by esterification, where two compounds, caffeic acid and quinic acid, are linked. For this reason, chlorogenic acid may have a higher content in fermented coffee [10,34,37,38].

In addition, epiphytic lactic acid bacteria and yeasts can protect these compounds by stabilizing the environment through the production of organic acids from the primary metabolism of growing microorganisms, such as lactic acid, which promotes the reduction of chlorogenic acid oxidation [39,40].

For trigonelline, a strong correlation (0.8) was observed between the yeasts and the P2 variety, as quantified using YPD agar. For caffeine, a correlation was observed only in variety A between lactic acid bacteria (0.83) and mesophilic bacteria (0.75). For variety CP, no correlations were observed between the microbial groups and the bioactive compounds (Supplementary Material—Figure S3).

The use of *S. cerevisiae* CA11 influenced the maximum accumulation of trigonelline and chlorogenic acid in the P2 (trigonelline: 0.07 mg·L^−1^; chlorogenic acid: 0.36 mg·L^−1^) and CA62 (trigonelline: 0.07 mg·L^−1^; chlorogenic acid: 0.27 mg·L^−1^) coffee varieties. Compared to using *K. lactis* B10, the concentration and accumulation of bioactive compounds depended on the coffee variety throughout the fermentation process. The trigonelline concentration was more significant for A (0.06 mg·L^−1^), P2 (0.07 mg·L^−1^), and CA62 (0.07 mg·L^−1^), and the concentration of chlorogenic acid was greater only in P2 (0.36 mg·L^−1^) and CP (0.33 mg·L^−1^). However, in the context of spontaneous fermentation, the P2 variety showed a reduction in the concentration of approximately 28% of these compounds throughout fermentation, resulting in coffee beans with lower caffeine concentrations (N: 0.07 mg·L^−1^; CA11: 0.08 mg·L^−1^; B10: 0.08 mg·L^−1^) and trigonelline (N: 0.05 mg·L^−1^; CA11: 0.07 mg·L^−1^; B10: 0.07 mg·L^−1^) (Supplementary Material—Table S2).

A hierarchical cluster analysis (HCA) was designed to support the multivariate analysis presented. In HCA, the samples are grouped into classes according to their proximity or similarity [41], and the results are shown as a dendrogram (Figure 2B). The samples were separated into three visible groups, in agreement with PCA and with the variability of the data analyzed using the Scott–Knott test. The treatments presented in the blue group represent the biochemical variation in the different coffee varieties during fermentation compared to the treatment without a starter culture, as evidenced by the accumulation of bioactive compounds. In the second group (noted in red), there was a separation of the treatments according to the use of the starter cultures at 24 and 48 h, so varieties A, P2, and CA62 had higher concentrations of these compounds than those in the blue group. The third group (noted in yellow) was separated according to the characteristics of the coffee varieties at the beginning of the fermentation process, where they had relatively high concentrations of sugars, such as glucose and fructose.

### 3.4. Evaluation of the Effect of Yeast Starter Cultures on the Volatile Compound Profile

The complexity of coffee flavor is mainly attributed to volatile compounds, which may be inherent to the coffee beans, as well as microbial metabolites resulting from the fermentation process [9,42]. Based on their odor quality, they can be classified into nine main odorant groups: (i) roasted, (ii) spicy, (iii) nutty/cocoa, (iv) sweet, (v) floral, (vi) fruity, (vii) acidic/fermented, (viii) green/vegetative, and (ix) other (including chemical and paper/mold odors). These are represented in the Specialty Coffee Association’s (SCA) Coffee Taster’s Flavor Wheel based on the World Coffee Survey’s Sensory Lexicon [1,43].

**Figure 5:**
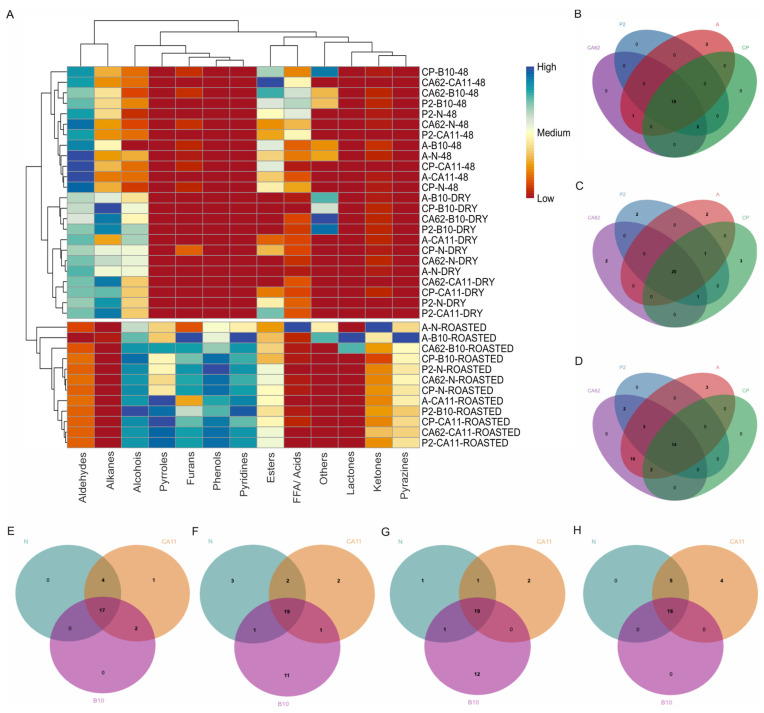
Heatmap of chemical classes of volatile compounds and grouping of coffee cultivars fermented using different inoculants (**A**): (48 h, dry and roasted). Venn diagram of volatile compounds as a function of inoculation and roasted coffee variety. (**B**): N; (**C**): CA11; (**D**): B10; (**E**): CP; (**F**): P2; (**G**): CA62; (**H**): A.

A total of 100 volatile compounds were detected in green and roasted coffee using SPME/GC–MS (Supplementary Material—Tables S3–S6). These compounds were grouped into 13 chemical classes: aldehydes (20), alkanes (9), ketones (6), phenols (1), alcohols (19), esters (10), furans (7), pyrazines (7), pyrroles (4), free fatty acids (FFA/acids) (9), pyridines (1), lactones (2), and other compounds (5) (Figure 5).

The starter cultures altered the profile of volatile compounds. In addition, the profile differed depending on the coffee variety evaluated. After fermentation (48 h), aldehydes predominated in green coffee, and the highest abundance was observed in coffee variety A (73.63%) and CP when subjected to fermentation by *S. cerevisiae* CA11. This behavior occurred probably due to the intense catabolism of sugars during the fermentation of these coffee varieties in the presence of *S. cerevisiae* CA11, as noted in Section 3.3 of this text (Figure 4B). The abundance of aldehydes decreased during fermentation, drying, and roasting, which indicates that processing influenced the volatile profile. In addition, using different metabolic pathways, the starter cultures contributed to the biotransformation of these compounds present in green coffee, which consequently enabled the production of distinct volatile compounds during roasting [30,44,45]. The most abundant compound at the end of fermentation was benzeneacetaldehyde, which gave the coffee a green, honey-like aroma. These aromatic nuances are considered green/vegetative and sweet [43,46].

Still highlighting the compounds in the fermented green coffee varieties, it was observed that esters were abundant only in the CA62 variety when *S. cerevisiae* CA11 was used as the starter culture. The presence of this chemical class is due to ester production through the metabolism of carbon and nitrogen during fermentation [47].

The roasting process triggers biochemical reactions that provide flavor and aroma to roasted coffee. The most predominant chemical classes in the roasted coffee varieties were alcohols, pyrroles, furans, phenols, and pyridines. Flavonoids such as furfurals, pyrazines, and pyrroles originate from the Maillard reaction through the precursors 3-deoxyosone and furans in the caramelization of sugars [48]. Natural fermentation resulted in 19 compounds common to all the roast coffee varieties evaluated. However, it was possible to observe that some compounds may be present exclusively depending on the coffee variety observed. Coffee variety A and CA62 have three unique compounds, pyrazine, 3-ethyl-2,5-dimethyl, and corylon, which give this variety nuances of cereals, bread, mushrooms, and potatoes, such as a burnt and toasted appearance. This type of sensory description leads the coffee to be considered a coffee without many sensory expressions (Figure 4B) [49].

In addition to the starter cultures influencing the volatile profile, differences in this variable were also observed between the coffee varieties studied. This variation may be linked to genetic differences in the coffee varieties used [50]. The CP coffee variety was able to accumulate specific compounds, such as 1-ethylpyrrole, 2-formylpyrrole, 1-furfurylpyrrole, and hexadecanoic acid (Figure 5G), which promoted the formation of fresh notes, vegetables, and a full-bodied toast. It was also possible to observe this behavior for the CA62 and P2 coffee variety, where 2-formylpyrrole was the only compound that was present in this variety and could bring out roasted and nutty notes, contributing to the construction of a coffee drink in the nutty and cocoa category (Figure 5F) [51].

When *K. lactis* B10 was used in the fermentation of the green beans, the roasted beans had greater uniqueness than *S. cerevisiae* CA11. This was due to the volatile compound precursors present at the end of fermentation, which were biotransformed to individualize the coffee varieties. The conversion of aldehyde starter cultures into higher alcohols after fermentation, through the activity of alcohol dehydrogenases, made it possible for this chemical group to be more abundant in the P2 (43.77%) and CP (41.50%) varieties [52]. This predominance is mainly due to furfuryl alcohol, constituting 39.62% of the P2 variety and 37.62% of CP. Furfuryl alcohol provides sweet and mild caramel notes to coffee (Figure 4A) [48].

The furans, in turn, were represented mainly by the presence of 5-hydroxymethylfurfural, which was greater in variety A when *K. lactis* B10 was used for fermentation (8.92%). The furfural content ranged between 0.23% (A) and 14.74% (CP), so its maximum predominance depended on the coffee variety and use of the starter culture. These compounds reinforce coffee’s sweet, woody, almond, and roasted flavors and are classified in the sweet category on the flavor wheel [51].

The content of pyrazines presented a variation of 6.69–18.70% among the studied coffee varieties. These compounds are mainly derived from Strecker’s degradation of α-amino acids during roasting. Seven pyrazines were found in the coffee samples fermented with *K. lactis* B10. The coffee variety A fermented by *K. lactis* B10 was the one with the higher content of pyrazines. Here, methylpyrazine (11.96%) had the greatest abundance among the identified pyrazines. All of them are considered potent coffee odorants, characterized by notes of nutty, cocoa, and roasted odors and, consequently, classified in the nutty/cocoa category [13,51]. Pyridines, pyrroles, and phenols were identified in all treatments and were less dominant, although their abundance was greater when starter cultures were present (Figure 5A).

Considering the individuality of fermentations in the presence of starter cultures, it was observed that of the 100 compounds identified, some volatiles were only present when starter cultures were used due to the production of secondary metabolites during fermentation, which can migrate to the beans and improve flavor [31] (Figure 5). 5-Methyl-2-furfuryl alcohol and n-hexadecanoic acid were produced exclusively in the presence of yeasts. Regarding fermentation using *S. cerevisiae* CA11, of the 22 common compounds, only CP exhibited the unique compound hexadecanoic acid (Figure 5C). This compound constitutes the lipid fraction of coffee, so its presence after roasting results from the fermentation of green coffee, the transesterification of triglycerides containing palmitic acid with ethanol, or the direct esterification of free palmitic acid with ethanol. This compound can impart fatty and sour nuances, forming part of the sour/fermented category [50,51].

In contrast to the use of *S. cerevisiae* CA11, the use *of K. lactis* B10 resulted in a smaller variety of volatile compounds in common at coffee varieties, with only 14 detected, and variety A accumulated 3 exclusive compounds of different classes, namely, 2-dodecenal, dodecane, and furyl ethyl ketone (Figure 5D). These compounds impart persistent fatty, citrus, and herbaceous nuances, categorized as green/vegetative [46].

The volatile profiles of the CA62 and A varieties fermented by *K. lactis* B10 were similar, as the presence of compounds such as 2,3-pentanedione, dihydro-2-methyl-3(2H)-furanone, 2-butylfuran, 2,5-dimethylpyrazine, β-angelica lactone, and δ-octalactone promoted sweet, nutty, and buttery fruity notes. This note was classified into fruity, sweet, and nutty/cocoa (Figure 5G,H). However, the presence of compounds such as 2-methyl butanoic acid, specific acid, and 5-methyl-2-furfuryl alcohol in CA62 increases the sensory perception of acidity. In contrast, in variety A, aldehydes, alkanes, and ketones add fruity and floral coffee aromas [30].

### 3.5. Sensory Profiles of Roasted Coffees

**Figure 6:**
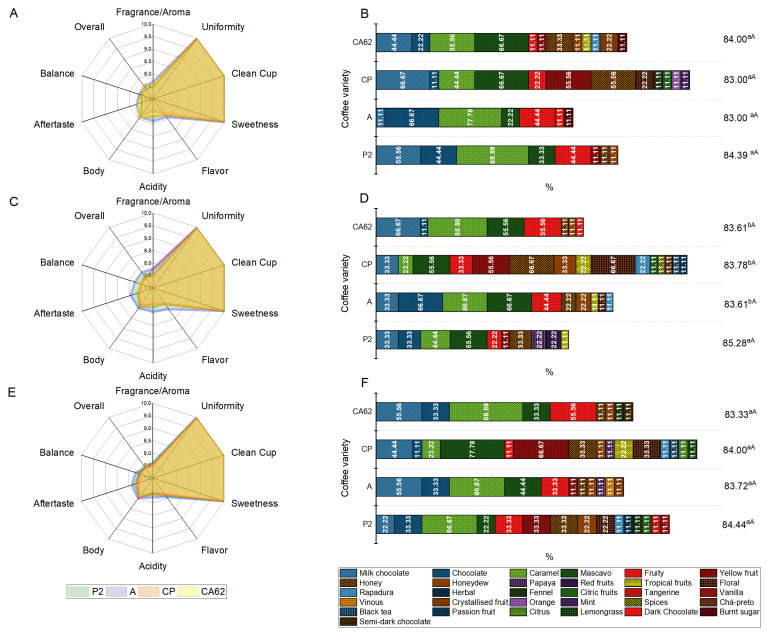
Sensory evaluation of the route means scores of the attributes of the fermented coffee cultivars evaluated using the SCAA excavation protocol (**A**): N; (**C**): CA11; (**E**): B10. Sensory descriptors and final scores of coffees with and without starter yeasts (**B**): N; (**D**): CA11; (**F**): B10. Mean ± standard deviation followed by lower case letters and upper case letters for the final score show statistical differences determined using the Scott–Knott test (*p* < 0.05).

The use of starter cultures provided different flavors and aromas. It increased the complexity of the beverage, as shown by the presence and accumulation of volatile compounds in the coffee varieties (Figure 5). This difference in sensory quality is caused by a change in the chemical composition of the coffee beans, which is highly influenced by microorganisms, enzymatic reactions, and environmental parameters such as temperature, presence/absence of oxygen, and processing time [18]. No statistical differences were detected in the flavor, body, aftertaste, or general attributes (*p* < 0.05). All treatments scored 10 for uniformity, clean cup, and sweetness. The scores of the other attributes are shown in Figure 6 and Table S7 in the Supplementary Material.

Compared with the other varieties, the coffee variety P2 had the greatest complexity regarding its aroma, acidity, and balance attributes, especially in the presence of *S. cerevisiae* CA11. In addition, for variety A, the starter culture had specific aromas that may reflect the quality and complexity of the culture, as well as the consumer preference. The presence of yeasts such as *M. caribbica*, *H. uvarum*, and *P. kluyveri* and bacteria such as *Bacillus albus* in the wet fermentation of these varieties promoted the modulation, differentiation, and enhancement of sensory descriptors in coffee, contributing to the aromatic robustness of the beverage [53].

Sensory descriptors such as chocolate nuance, milk chocolate, caramel, and browned aromas were noted in all treatments (Figure 6; Supplementary Material—Table S7). In addition, all samples presented fruity notes (red or yellow, tropical or candied fruits) and sweetness since the treatments presented the descriptors honey, rapadura, and molasses. The CA62 variety had a greater presence of caramel aroma than the other varieties, regardless of the starter yeast used. In addition, 2-formylpyrrole is associated with roasted and nutty nuances, reinforcing descriptors such as “dark chocolate” and “cocoa” in this coffee variety when *S. cerevisiae* CA11 was used as the starter culture [52].

*K. lactis* B10 allowed the CP and P2 coffee varieties to be described as a coffee with notes of citrus, lemongrass, and lemon with honey and yellow fruit. This description is possible due to the presence of aldehydes, alkanes, and ketones, reinforcing the fresh and acidic aromatic profile of these coffee varieties due to the presence of compounds such as 5-methyl-2-furfuryl alcohol and n-hexadecanoate (Figure 4A). The floral descriptor was present only in the treatments where yeasts were used as starter cultures and in the varieties A, P2, and CP, whereas the latter variety had a greater presence of this descriptor when *S. cerevisiae* CA11 was used in the fermentation process. This variety also had unique descriptors, such as lemon with honey, passion fruit, black tea, and spices, arising from the accumulation of unique volatile compounds attributed to the presence of benzaldehyde, for example (Figure 5E and Figure 6) [51,52]. Other descriptors including vanilla and tangerine (P2-B10), orange and mint (CP-N), dark chocolate (CA62-CA11e AN), and semisweet chocolate (CA62-B10) were also present.

In general, the beverages of the studied coffee varieties had similar total scores (*p* > 0.05), as evaluated by Q-graders (Figure 6B,D,F). Therefore, the treatments were considered very good specialty coffees, as they obtained a final score higher than 83, except for the P2 variety when fermented in the presence of *S. cerevisiae* CA11, which obtained a score above 85 and was considered an excellent coffee [54]. The final score and sensory characteristics are gaining increasing importance in the purchase decision of specialty coffee connoisseurs due to changes in consumer behavior toward coffees [53].

## 4. Conclusions

The inoculation of starter yeasts in the fermentation process significantly and differently impacted the quality of the coffee varieties. The presence of *S. cerevisiae* CA11 gave the coffees fruity and floral notes, especially in the MGS Catucai Pioneira (CP) and MGS Paraíso 2 (P2) varieties. Descriptors of yellow fruit and honey were present in greater abundance when the coffees were fermented with *K. lactis* B10. These differences were observed mainly due to the different carbohydrate consumption profiles during fermentation, which was mainly attributed to the different metabolisms of the yeasts studied. Given the results and the definition of specialty coffees, the use of starter yeasts is a strategy to improve quality and to obtain coffees with unique characteristics.

## Data Availability

The original contributions presented in the study are included in the article; further inquiries can be directed to the corresponding author.

## Supplementary Materials

The following supporting information can be downloaded at: https://www.mdpi.com/article/10.3390/foods14010111/s1, Table S1: Standardization/validation parameters for chromatographic methods; Figure S1: High-performance liquid chromatography of fermented coffees. (A) Chromatogram of the standard solution (0.075 mg mL^−1^) and sample (bean) of (T) trigonelline, (A) chlorogenic acid, and (C) caffeine; (B) Chromatogram of the standard solution (3.0 mg mL^−1^) and sample (bean) of (G) glucose and (F) fructose; Table S2: Carbohydrates content and bioactive compounds during fermentation of coffee cultivars fermented with different types of inoculum; Figure S2: Variables contributing to the construction of the dimensions in the principal component analysis (PCA) of coffees fermented with different yeast starters; Table S3: Volatile compounds (%) and sensory descriptors determined using HS-SPME/GC-MS in the Catuaí Amarelo coffee variety (CA62) fermented with different inoculums; Table S4: Volatile compounds (%) and sensory descriptors determined using HS-SPME/GC-MS in the MGS Paraiso 2 (P2) coffee variety fermented with different inoculums; Table S5: Volatile compounds (%) and sensory descriptors determined using HS-SPME/GC-MS in the MGS Ametista coffee variety (A) fermented with different inoculums; Table S6: Volatile compounds (%) and sensory descriptors determined using HS-SPME/GC-MS in the coffee variety MGS Catuaí Pioneira (CP) fermented with different inoculums; Table S7: Sensory evaluation notes on coffee drinks from genetic varieties of arabica coffee subjected to fermentation; Figure S3: Pearson correlation of different arabica coffee cultivars fermented using yeast starters (A): CA62; (B): A; (C): P2; (D): CP)-(*) Correlation is significant at a 0.05 level; (**) Correlation is significant at a 0.01 level; (***) Correlation is significant at a 0.001 level.
