# Supplementary material for: Kluyveromyces lactis and Saccharomyces cerevisiae for Fermentation of Four Different Coffee Varieties
Source: Foods. 2025 Jan 3;14(1):111. doi: 10.3390/foods14010111 (PMC11719620; doi:10.3390/foods14010111)

SUPPLEMENTARY MATERIAL

Kluyveromyces lactis and *Saccharomyces cerevisiae* for Fermentation of Four Different Coffee Varieties

Danilo José Machado de Abreu [1,2], Denis Henrique Silva Nadaleti [3], Rafaela Pereira Andrade [1], Tamara Leite dos Santos [1], Dérica Gonçalves Tavares [4], Cesar Elias Botelho [3], Mário Lúcio Vilela de Resende [1,3] and Whasley Ferreira Duarte [1,2,*]

[1] Instituto Nacional de Ciência e Tecnologia do Café (INCT), Lavras CEP 37203-202, MG, Brazil; danilo.mabreu@gmail.com (D.J.M.d.A.); rafaelaandrade1210@gmail.com (R.P.A.); tamaraleitesantos@gmail.com (T.L.d.S.)
[2] Biology Department, Federal University of Lavras (UFLA), Lavras CEP 37203-202, MG, Brazil
[3] Empresa de Pesquisa Agropecuária de Minas Gerais (EPAMIG), Lavras CEP 37203-202, MG, Brazil; denis.nadaleti@epamig.br (D.H.S.N.); cesarbotelho@epamig.br (C.E.B.)
[4] Department of Biology, University of Louisville, Louisville, KY 40208, USA; derica.goncalvestavares@louisville.edu
* Correspondence: whasleyduarte@ufla.br

Table S1 - Standardisation/validation parameters for chromatographic methods.

Parameters	Trigonelline	Chlorogenic acid	Caffeine	Glucose	Fructose
**B**	3E7	2E7	6E7	353525	387207
**A**	38122	21533	77425	-4279	-970
**r^2**	0,999	0,999	0,999	0,999	0,999
**LD (mg mL^{-1})**	0,012	0,008	0,009	0,02	0,06
**LQ (mg mL^{-1})**	0,041	0,027	0,032	0,09	0,20
**Recovery (%)**	82 a 99	80 a 101	89 a 104	70 a 88	71 a 89
**CV (%)**	0,41 a 1,12	0,23 a 1,19	0,21 a 1,28	0,11 a 1,43	0,84 a 2,63

Figure S1 – High-performance liquid chromatography of fermented coffees. A) Chromatogram of the standard solution (0.075 mg mL-1) and sample (bean) of (T) trigonelline, (A) chlorogenic acid and (C) caffeine; B) Chromatogram of the standard solution (3.0 mg mL-1) and sample (bean) of (G) glucose and (F) fructose.

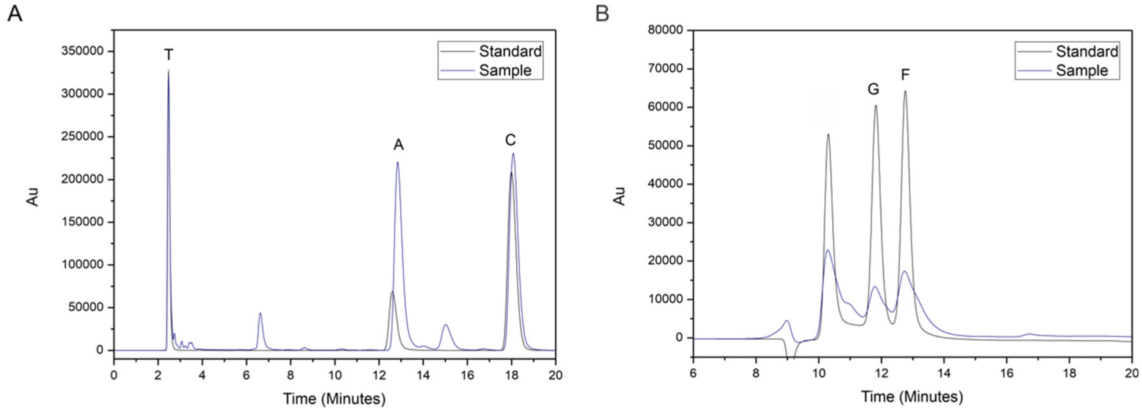

Table S2. Carbohydrates content and bioactive compounds during fermentation of coffee cultivars fermented with different types of inoculum.[1]

Cultivars	N			S. cerevisiae CA11			K. lactis B10		
	0	24	48	0	24	48	0	24	48
	Caffeine (mg. L^{-1})								
CP	0.06±0.00^{bA*}	0.08±0.00^{aA*}	0.08±0.00^{aA*}	0.07±0.03^{aA*}	0.08±0.00^{aA*}	0.08±0.00^{aA*}	0.06±0.00^{bA*}	0.08±0.00^{aA*}	0.08±0.00^{aA*}
CA62	0.07±0.00^{aA*}	0.08±0.00^{aA*}	0.08±0.00^{aA*}	0.07±0.00^{bA*}	0.08±0.00^{aA*}	0.09±0.00^{aA*}	0.07±0.00^{bA*}	0.07±0.00^{bA*}	0.09±0.00^{aA*}
A	0.06±0.00^{bA*}	0.08±0.00^{aA*}	0.08±0.00^{aA*}	0.07±0.00^{aA*}	0.08±0.00^{aA*}	0.09±0.00^{aA*}	0.07±0.00^{bA*}	0.08±0.00^{aA*}	0.09±0.00^{aA*}
P2	0.07±0.00^{aA*}	0.07±0.00^{aA*}	0.07±0.00^{aB*}	0.02±0.00^{bB**}	0.08±0.04^{aA*}	0.08±0.00^{aA*}	0.06±0.00^{bA*}	0.08±0.00^{aA*}	0.08±0.00^{aA*}
	Chlorogenic acid (mg. L^{-1})								
CP	0.25±0.01^{bA**}	0.33±0.00^{aA*}	0.33±0.01^{aA*}	0.29±0.00^{aA*}	0.23±0.01^{bB**}	0.24±0.01^{bB**}	0.21±0.00^{bA**}	0.25±0.01^{cB**}	0.33±0.01^{aA*}
CA62	0.23±0.00^{aB*}	0.25±0.01^{aB*}	0.25±0.00^{aB*}	0.22±0.01^{bB*}	0.22±0.01^{bB*}	0.27±0.01^{aB*}	0.23±0.01^{bA*}	0.23±0.01^{bB*}	0.28±0.02^{aB*}
A	0.17±0.01^{cC*}	0.21±0.00^{bC**}	0.26±0.00^{aB*}	0.20±0.00^{bB*}	0.21±0.01^{bB**}	0.28±0.00^{aB*}	0.18±0.01^{bB*}	0.28±0.00^{aA*}	0.28±0.01^{aB*}
P2	0.20±0.01^{aB*}	0.31±0.00^{aA**}	0.33±0.01^{aA*}	0.28±0.01^{bA*}	0.35±0.03^{aA*}	0.36±0.04^{aA*}	0.27±0.02^{cA*}	0.31±0.00^{bA**}	0.36±0.01^{aA*}
	Trigonelline (mg. L^{-1})								
CP	0.04±0.00^{bC*}	0.04±0.00^{bA*}	0.06±0.00^{aB*}	0.05±0.00^{aB*}	0.05±0.00^{aC**}	0.05±0.00^{aC*}	0.05±0.00^{bB*}	0.06±0.00^{aA*}	0.07±0.00^{aB*}
CA62	0.06±0.00^{aA*}	0.06±0.00^{aA*}	0.06±0.00^{aA**}	0.06±0.00^{bA*}	0.06±0.00^{bB*}	0.07±0.00^{aA*}	0.06±0.00^{bA*}	0.06±0.00^{bA*}	0.07±0.00^{aA*}
A	0.05±0.00^{bC**}	0.06±0.00^{aA*}	0.06±0.00^{aA**}	0.06±0.00^{aA*}	0.06±0.00^{aB*}	0.06±0.00^{aB**}	0.04±0.00^{bB**}	0.04±0.00^{bB**}	0.06±0.00^{aA*}
P2	0.07±0.00^{cB*}	0.06±0.00^{bA**}	0.05±0.00^{aB**}	0.06±0.00^{bA**}	0.07±0.00^{aA*}	0.07±0.00^{aA*}	0.05±0.00^{cA**}	0.06±0.00^{bA**}	0.07±0.00^{aA*}
	Glucose (g. L^{-1})								
CP	0.73±0.05^{aB*}	0.25±0.08^{bA*}	0.10±0.00^{cA*}	0.78±0.06^{aA*}	0.18±0.01^{bA*}	0.07±0.00^{bA*}	0.82±0.04^{aA*}	0.24±0.08^{bA*}	0.11±0.00^{bA*}
CA62	0.74±0.15^{aB*}	0.15±0.01^{bA*}	0.12±0.03^{bA*}	0.56±0.32^{aB*}	0.13±0.03^{bA*}	0.08±0.00^{bA*}	0.62±0.15^{aB*}	0.25±0.03^{bA*}	0.13±0.02^{bA*}
A	0.93±0.07^{aA*}	0.19±0.05^{bA**}	0.10±0.01^{bA*}	0.89±0.01^{aA*}	0.12±0.02^{bA**}	0.10±0.01^{bA*}	0.67±0.08^{aB**}	0.30±0.01^{bA*}	0.10±0.01^{cA*}
P2	0.52±0.07^{aC*}	0.21±0.01^{bA*}	0.07±0.00^{bA*}	0.59±0.09^{aB*}	0.12±0.32^{bA*}	0.07±0.00^{bA*}	0.45±0.08^{aC*}	0.20±0.04^{bA*}	0.11±0.02^{bA*}
	Fructose (g. L^{-1})								
CP	1.53±0.12^{aB*}	0.66±0.12^{bA*}	0.38±0.02^{bA*}	1.67±0.10^{aB*}	0.36±0.04^{bA*}	0.19±0.03^{bA*}	1.78±0.07^{aA*}	0.52±0.15^{bA*}	0.27±0.00^{bA*}
CA62	1.26±0.29^{aC*}	0.45±0.03^{bA*}	0.34±0.05^{bA*}	1.09±0.53^{aB*}	0.37±0.05^{bA*}	0.21±0.03^{bA*}	1.15±0.18^{aC*}	0.58±0.08^{bA*}	0.35±0.08^{bA*}
A	1.86±0.14^{aA*}	0.52±0.13^{bA*}	0.41±0.03^{bA*}	1.81±0.03^{aA*}	0.31±0.05^{bA*}	0.28±0.03^{bA*}	1.35±0.20^{aB**}	0.65±0.06^{bA*}	0.30±0.04^{cA*}
P2	0.98±0.15^{aC*}	0.60±0.03^{bA*}	0.30±0.02^{bA*}	1.04±0.20^{aA*}	0.29±0.47^{bA*}	0.20±0.02^{bA*}	0.80±0.19^{aD*}	0.45±0.09^{bA*}	0.30±0.07^{bA*}

[1] Means ± standard deviation followed by different lower case letters horizontally, different upper case letters vertically, single, double or triple asterisks vertically show statistical differences using the Scott-Knott test ($p<0.05$). CA62: Catuaí Amarelo IAC62; A: MGS Amestista; P2: MGS Paraíso 2; CP: MGS Catuaí Pioneira.

Figure S2. Variables contributing to the construction of the dimensions in the principal component analysis (PCA) of coffees fermented by different yeast starters.

A

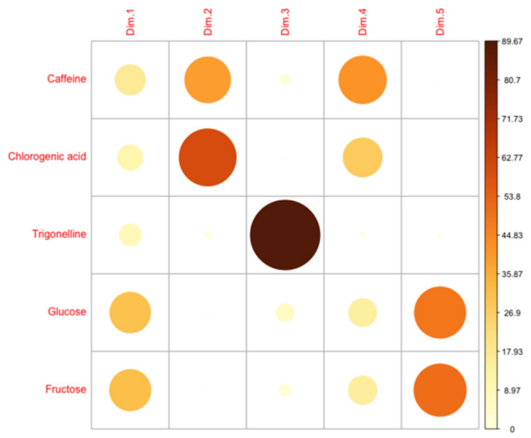

B

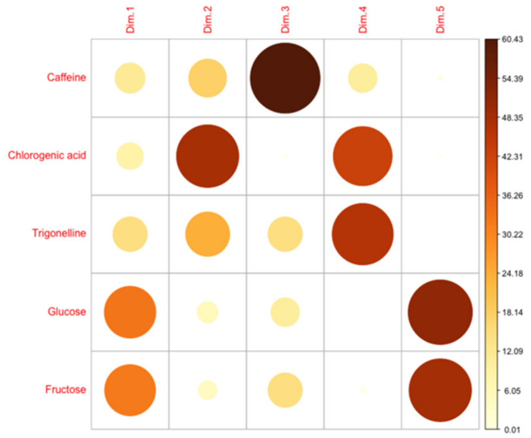

C

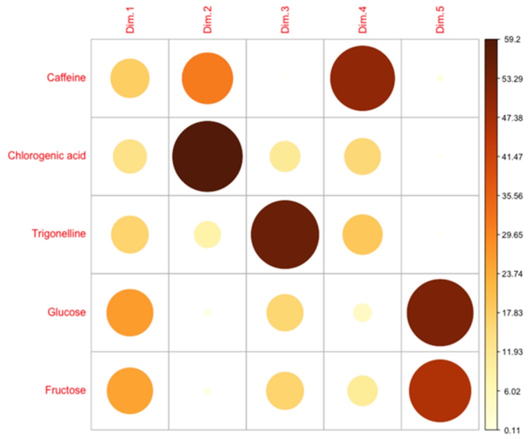

Table S3. Volatile compounds (%) and sensory descriptors determined by HS-SPME/GC-MS in the Catuaí Amarelo coffee variety (CA62) fermented with different inoculums[1].

No.	Class	Volatile compounds	Retention Time	Retention Index	Odor descriptors	N			CA11			B10		
	Aldehydes					48 hours	Dry	Roasted	48 hours	Dry	Roasted	48 hours	Dry	Roasted
1		3-Methylbutanal	3,47	651	Ethereal, aldehydic, chocolate, peach, fatty	7,20	0,87	0,00	5,59	0,63	0,00	4,44	0,51	0,00
2		2-Methylbutanal	3,610	657	Musty, cocoa, phenolic, coffee, nutty, malty, fermented, fatty alcoholic	2,24	1,02	0,00	0,79	0,93	0,00	1,23	0,64	0,00
3		Hexanal	7,542	801	Green, fatty, leafy, vegetative, fruity and clean with a woody nuance	3,15	5,06	0,00	2,26	5,92	0,00	2,90	0,00	0,00
4		Heptanal	12,519	902	Fresh, aldehydic fatty, green, herbal, cognac, ozone	0,00	0,50	0,00	0,00	0,00	0,00	0,00	0,39	0,00
5		2-Heptenal, (E)	15,536	957	Green	0,72	1,89	0,00	0,00	1,35	0,00	0,29	1,11	0,00
6		Octanal	18,044	1003	Waxy, green, citrus, aldehydic and floral with a sweet, fatty, coconut nuance	0,66	1,05	0,00	0,00	1,01	0,00	0,70	0,00	0,00
7		2-Octenal, (E)	20,996	1058	Fatty	1,28	0,00	0,00	0,00	1,09	0,00	0,00	0,90	0,00
8		Nonanal	23,416	1103	Waxy, aldehydic, citrus, with a fresh slightly green lemon peel like nuance, and a cucumber fattiness	10,20	19,08	0,00	13,36	20,96	0,00	10,56	22,08	0,00
9		2-Nonenal, (E)	26,230	1158	Green, cucumber, aldehydic, fatty with a citrus nuance	3,08	2,84	0,00	2,33	2,85	0,00	1,99	0,00	0,00
10		2-Butenal, 2-methyl	6,945	780	Diffusive, pungent, green, ethareal, sharp penetrating, nutty and anisic	0,00	1,02	0,00	0,00	0,00	0,00	0,57	0,97	0,00
11		Decanal	28,5	1203	Sweet, aldehydic, orange, waxy and citrus rind	5,05	4,21	0,00	6,97	5,65	0,00	5,26	4,78	0,00
12		Methional	12,77	903	Creamy tomato, potato skin and French fry, yeasty, bready, limburger cheese with a savory meaty brothy nuance	0,00	0,00	0,00	0,99	0,00	0,00	0,00	0,00	0,00
13		Benzeneacetaldehyde	20,172	1042	Green, honey	29,68	10,43	0,00	22,82	9,25	0,00	20,12	6,77	0,00
14		Benzaldehyde	15,705	960	Sharp, sweet, bitter, almond, cherry	6,08	5,96	0,00	5,41	4,21	0,00	4,76	5,58	0,00
15		Furfural	9,056	832	Sweet, woody, almond, bread baked	0,00	0,00	14,74	0,00	0,00	12,88	0,00	0,51	11,64
16		2-Butenal, 3-methyl	7,056	784	Sweet, fruity, green, nutty, cherry	ND	ND	ND	0,76	0,00	0,00	ND	ND	ND
17		14-Heptadecenal	49,861	1867	-	ND	ND	ND	ND	ND	ND	ND	ND	ND
18		2-Dodecenal, (E)-	23,428	1103	-	ND	ND	ND	ND	ND	ND	ND	ND	ND
19		2-Propenal	19,246	1025	-	ND	ND	ND	ND	ND	ND	ND	ND	ND
20		Prenal	7,055	784	Sweet, fruity, pungent, brown and nutty with an almond and cherry nuance	ND	ND	ND	ND	ND	ND	ND	ND	ND
		TOTAL				69,35	53,93	14,74	61,29	53,86	12,88	52,82	44,25	11,64
	Alkanes													
21		Dodecane	28,221	1197	-	1,21	3,52	0,00	0,00	6,04	0,00	1,30	5,77	0,00
22		Tetradecane	37,398	1398	-	4,79	9,20	0,00	5,53	11,74	0,00	5,12	10,15	0,00
23		Hexadecane	41,601	1498	-	0,00	1,84	0,00	0,00	3,27	0,00	1,82	5,09	0,00
24		4,7-Dimethylundecane	41,591	1498	-	ND	ND	ND	ND	ND	ND	1,19	0,00	0,00
25		3,3-Dimethylhexane	31,757	1272	-	ND	ND	ND	ND	ND	ND	ND	ND	ND
26		Phenethyl octanoate	34,364	1329	-	ND	ND	ND	ND	ND	ND	ND	ND	ND
27		2,2-Dimethylbutane	39,939	1458	-	ND	ND	ND	ND	ND	ND	ND	ND	ND
28		Decane	34,028	1321	-	ND	ND	ND	ND	ND	ND	ND	ND	ND
29		5-Octadecene, (E)	37,098	1391	-	ND	ND	ND	ND	ND	ND	ND	ND	ND
		TOTAL				6,00	14,56	0,00	5,53	21,05	0,00	9,44	21,01	0,00
	Ketones													
30		Acetophenone	21,325	1064	Sweet, pungent, hawthorn, mimosa, almond, acacia, chemical	0,33	0,47	0,00	0,00	0,00	0,90	0,45	0,40	0,00
31		1-Hydroxy-2-butanone	6,337	757	Coffee, sweet, brown, coffee, musty, grain- and pyrazine-like with malt and butterscotch nuances	0,00	0,00	1,03	0,00	0,00	1,13	0,00	0,00	1,02
32		1-Acetoxy-2-butanone	16,032	966	-	0,00	0,00	0,64	0,00	0,00	0,64	0,00	0,00	0,73
33		4-Hydroxy-3-hexanone	7,607	802	-	0,00	0,00	0,31	0,00	0,00	0,32	0,00	0,00	0,28
34		2,3-Pentanedione	4,194	678	Buttery, nutty, toasted, caramellic, diacetyl and acetoin notes	ND	ND	ND	ND	ND	ND	0,00	0,00	0,16
35		Isobutyl ketone	16,25	970	-									
		TOTAL				0,33	0,47	1,97	0,00	0,00	2,99	0,45	0,40	2,20
	Phenol													
36		2-Methoxy-4-vinylphenol	33,528	1310	Sweet, spicy, clove carnation, phenolic, peppery, smoky, woody, powdery	0,00	0,00	9,95	0,00	0,00	9,93	0,00	0,00	7,33
		TOTAL				0,00	0,00	9,95	0,00	0,00	9,93	0,00	0,00	7,33
	Alcohois													
37		Isopentyl alcohol	5,199	715	Fermented, fusel, alcoholic, pungent, etherial, cognac, fruity, banana, molasses	0,00	1,76	0,00	1,72	0,45	0,00	0,00	0,75	0,00
38		Prenyl alcohol	6,667	770	Sweet, fruity, alcoholic with a green nuance	0,44	0,00	0,00	0,45	0,00	0,00	0,00	0,50	0,00
39		Furfuryl alcohol	10,558	862	Alcoholic chemical, musty, sweet, caramel, bread, coffee	0,00	0,00	34,98	0,00	0,00	34,16	0,00	0,00	34,70
40		1-Hexanol	10,945	870	Pungent, etherial, fusel oil, fruity and alcoholic, sweet with a green top note	0,00	3,26	0,00	ND	ND	ND	ND	ND	ND
41		1-Octen-3-ol	16,843	981	Fresh, fungal, fruity, genuine, mushroom, earthy, violet, melon, humus	1,99	3,15	0,00	1,58	1,98	0,00	1,24	2,71	0,00
42		Benzyl alcohol	19,761	1035	Floral, rose, phenolic, balsamic	5,27	9,42	0,00	3,84	9,69	0,00	4,99	10,78	0,00
43		1-Octanol	21,754	1072	Waxy, green, orange, aldehydic, rose, mushroom	0,00	1,63	0,00	1,05	2,17	0,00	0,56	2,26	0,00
44		Phenylethyl Alcohol	23,811	1111	Floral, rose, dried rose	3,77	8,57	0,00	5,59	4,37	0,00	3,36	7,21	0,00
45		Maltol	23,847	1112	Sweet, caramellic, cotton, candy, jammy fruity, bread baked	0,00	0,00	3,29	0,00	0,00	3,92	0,00	0,00	3,67
46		Ethylhexanol	19,514	965	-	ND	ND	ND	0,00	1,53	0,00	ND	ND	ND
47		5-Methyl-2-furfuryl alcohol	15,494	956	-	ND	ND	ND	ND	ND	ND	0,00	0,00	0,35
48		2-Hexenol	19,498	1030	Fresh fatty green, fruity, vegetative, with leafy and herbal nuances	ND	ND	ND	ND	ND	ND	0,48	0,00	0,00
49		1-Heptanol, 6-methyl	21,772	1072	-	ND	ND	ND	ND	ND	ND	0,51	0,00	0,00

50		1-Dodecanol	44,651	1576	Earthy, soapy, waxy, fatty, honey, coconut	ND	ND	ND	ND	ND	ND	0,52	0,00	0,00
51		Z-2-Dodecenol	49,856	1768	-	ND	ND	ND	ND	ND	ND	0,00	0,76	0,00
52		3-Buten-2-ol	33,727	1314	-	ND	ND	ND	ND	ND	ND	ND	ND	ND
53		Isoamyl alcohol	5,243	717	Fusel, alcoholic, pungent, etherial, cognac, fruity, banana and molasses	ND	ND	ND	ND	ND	ND	ND	ND	ND
54		2-Buten-1-ol, propanoate	7,44	798	-	ND	ND	ND	ND	ND	ND	ND	ND	ND
55		1-Heptanol	26,938	1172	-									
		TOTAL			-	11,46	27,79	38,27	14,23	20,19	38,09	11,66	24,97	38,72
	Ester													
56		Methyl salicylate	27,805	1189	Wintergreen, minty	1,01	0,00	0,00	ND	ND	ND	ND	ND	ND
57		Undecyl acetate	22,644	1088	Oily, clean, waxy	0,00	0,00	0,70	0,00	0,00	0,71	ND	ND	ND
58		Furfuryl acetate	17,505	993	Sweet fruity, banana, horseradish	0,00	0,00	1,71	0,00	0,00	1,65	0,00	0,00	1,65
59		Ethyl isovalerate	10,068	852	Sweet, diffusive, estry, fruity, sharp, pineapple, apple, green and orange	ND	ND	ND	3,27	0,00	0,00	ND	ND	ND
60		Ethyl phenacetate	30,212	1239	Floral honey, rosy with balsamic dark chocolate and cocoa notes with anisic black licorice nuances	ND	ND	ND	1,25	0,00	0,00	0,75	0,00	0,00
61		Propyl propionate	7,403	797	Sharp, chemical, pungent with sweet fruity lift notes	ND	ND	ND	ND	ND	ND	0,37	0,00	0,00
62		Ethylmethylacetic acid	11,581	883	Etherial, fruity, sweet, grape and rum-like	ND	ND	ND	ND	ND	ND	1,21	0,00	0,00
63		5-Hexenyl propionate	19,474	1029	Green, fruity apple and pear pulp with creamy and powdery nuances	ND	ND	ND	ND	ND	ND	0,65	0,00	0,00
64		Ethyl hexadecanoate	56,627	2200	Waxy, fruity, creamy and milky with a balsamic nuance	ND	ND	ND	ND	ND	ND	0,59	0,00	0,00
65		Ethylhexanol	19,47	1029	-									
		TOTAL				1,01	0,00	2,41	4,52	0,00	2,36	3,58	0,00	1,65
	Furan													
66		5-Hydroxymethylfurfural	29,866	1232	Fatty, buttery, musty, waxy, caramellic	0,00	0,00	5,12	0,00	0,00	5,40	0,00	0,00	4,87
67		Furan, 2-pentyl	17,295	989	Fruity, green, earthy, beany, vegetable, metallic	1,79	0,00	0,00				1,68	0,00	0,00
68		Furfural, 5-methy	15,82	962	Spicy, caramellic, maple	0,00	0,00	11,75	0,00	0,00	11,69	0,00	0,00	11,16
69		2- Acetylfuran	12,943	910	Sweet, balsamic, almond, cocoa, caramellic, coffee	0,00	0,00	1,47	0,00	0,00	1,37	0,00	0,00	1,49
70		Dihydro-2-methyl-3(2H)-furanone	7,878	808	Sweet and solvent-like with a brown, rummy and nut-like nuance	ND	ND	ND	ND	ND	ND	0,00	0,00	1,06
71		2-Butylfuran	15,267	952	Fruity, winey, sweet, spicy	ND	ND	ND	ND	ND	ND	0,00	0,00	0,03
72		2-Amylfuran	17,33	990	-	ND	ND	ND	ND	ND	ND			
		TOTAL				1,79	0,00	18,34	0,00	0,00	18,46	1,68	0,00	18,60
	Pyrazines													
73		Pyrazine, 3-ethyl-2,5-dimethyl	21,97	1076	Potato, cocoa, roasted, nutty	0,00	0,00	0,82	ND	ND	ND	ND	ND	ND
74		2-Ethyl-6-methylpyrazine	17,795	998	Roasted potato	0,00	0,00	0,65	0,00	0,00	0,58	0,00	0,00	1,29
75		2-Ethyl-3-methylpyrazine	18,009	1002	Nutty, peanut, musty, corn raw, earthy, bready	0,00	0,00	1,73	0,00	0,00	1,70	0,00	0,00	0,89
76		Furfuryl formate	12,739	906	Ethereal	0,00	0,00	0,62				0,00	0,00	0,53
77		Methylpyrazine	8,648	823	Nutty, cocoa, roasted, chocolate, peanut green	0,00	0,00	4,42	0,00	0,00	4,01	0,00	0,00	4,74
78		Pyrazine	5,281	718	Sour, fishy, ammoniacal	0,00	0,00	0,49	0,00	0,00	0,55	0,00	0,00	0,60
79		2,5-Dimethylpyrazine	13,224	915	-	ND	ND	ND	ND	ND	ND	0,00	0,00	1,28
		TOTAL				0,00	0,00	8,72	0,00	0,00	6,84	0,00	0,00	9,32
	Pyrrole													
80		2-Acetylpyrrole	21,62	1069	Musty, nut, skin, cherry maraschino, cherry coumarinic, licorice, bready, walnut bready	0,00	0,00	1,92	0,00	0,00	2,15	0,00	0,00	2,26
81		1- Ethylpyrrole	18,822	959	Burnt	ND	ND	ND	0,00	0,00	1,13	0,00	0,00	1,46
82		2 Formylpyrrole	19,045	961	Musty, beefy, coffee	ND	ND	ND	0,00	0,00	1,01	ND	ND	ND
83		1 Furfurylpyrrole	27,138	1152	Vegetative, careal, bready, radish, mushroom and potato nuances	ND	ND	ND	0,00	0,00	0,50	0,00	0,00	0,44
		TOTAL				0,00	0,00	1,92	0,00	0,00	4,79	0,00	0,00	4,16
	FFA/Acids													
84		Hexanoic acid	17,283	989	Sour, fatty, sweaty, cheesy	0,71	1,35	0,00	0,00	1,08	0,00	0,05	2,42	0,00
85		Senecioic acid	12,772	907	Milky, roasted, burnt	0,86	0,00	0,00				0,68	0,00	0,21
86		Butanoic acid, 2-methyl	11,426	880	Pungent acidic cheesy roquefort cheese cheesy	1,27	1,02	0,00	2,13	0,00	0,00	0,00	0,00	0,13
87		Isovaleric acid	10,081	853	Sour, sweaty ,cheesy, tropical	7,23	0,89	1,34	12,30	3,83	0,65	14,53	1,98	0,69
88		Propanoic acid	4,856	703	Pungent acidic, cheesy, vinegar	0,00	0,00	0,94	0,00	0,00	0,89	0,00	0,00	1,16
89		3-Methylvaleric acid	10,06	852	Animalic, sharp acidic cheesey, green with a fruity sweaty nuance	ND	ND	ND	ND	ND	ND	2,61	0,00	0,00
90		n-Hexadecanoic acid	56,204	2173	-	ND	ND	ND	ND	ND	ND	0,00	0,00	0,22
91		Butanoic acid	12,553	902	-	ND	ND	ND	ND	ND	ND			
92		Nonanoic acid	31,756	1272	-	ND	ND	ND	ND	ND	ND			
		Total			-	10,06	3,25	2,28	14,43	4,91	1,54	17,87	4,39	2,40
	Pyridines													
93		Pyridine	5,689	733	Nutty	0,00	0,00	1,38	0,00	0,00	1,48	0,00	0,00	1,50
		TOTAL			-	0,00	0,00	1,38	0,00	0,00	1,48	0,00	0,00	1,50
	Lactones													
94		beta.-Angelica lactone	14,458	937	-	ND	ND	ND	ND	ND	ND	0,00	0,00	0,15
95		Delta.-Octalactone	13,979	928	Sweet, coconut, creamy, coumarin and lactonic with a green, fatty nuance	ND	ND	ND	ND	ND	ND	0,00	0,00	0,18
		TOTAL				0,00	0,00	0,00	0,00	0,00	0,00	0,00	0,00	0,33
	Other													
96		Betula oil	27,805	1189	Sweet, methyl salicylate, aromatic, spicy and herbal with a phenolic nuance	ND	ND	ND	ND	ND	ND	0,67	2,13	0,00
97		Corylon	19,34	1104	-	ND	ND	ND	ND	ND	ND			
98		Linalol	23,178	1100	-	ND	ND	ND	ND	ND	ND			
99		Furyl ethyl ketone	18,229	1006	-	ND	ND	ND	ND	ND	ND	ND	ND	ND
100		Isoamyl nitrite	31,709	1271	-	ND	ND	ND	ND	ND	ND	ND	ND	ND
		TOTAL				0,00	0,00	0,00	0,00	0,00	0,00	0,67	2,13	0,00

[1]ND means that the compound was not detected. All odors descriptor were find on http://www.thegoodscentscompany.com/.

Table S4. Volatile compounds (%) and sensory descriptors determined by HS-SPME/GC-MS in the MGS Paraiso 2 (P2) coffee variety fermented with different inoculums[1].

No.	Class	Volatile compounds	Retention Time	Retention Index	Odor descriptors	N			CA11			B10		
	Aldehydes					48 hours	Dry	Roasted	48 hours	Dry	Roasted	48 hours	Dry	Roasted
1		3-Methylbutanal	3,47	651	Ethereal, aldehydic, chocolate, peach, fatty	5,77	0,44	0,00	5,59	0,63	0,00	4,67	0,48	0,00
2		2-Methylbutanal	3,610	657	Musty, cocoa, phenolic, coffee, nutty, malty, fermented, fatty alcoholic	1,36	0,43	0,00	0,79	0,93	0,00	1,29	0,60	0,00
3		Hexanal	7,542	801	Green, fatty, leafy, vegetative, fruity and clean with a woody nuance	2,05	4,37	0,00	2,26	5,92	0,00	1,78	4,11	0,00
4		Heptanal	12,519	902	Fresh, aldehydic fatty, green, herbal, cognac, ozone	ND	ND	ND	ND	ND	ND	0,00	5,97	0,00
5		2-Heptenal, (E)	15,536	957	Green	0,00	1,39	0,00	0,00	1,35	0,00	0,30	1,05	0,00
6		Octanal	18,044	1003	Waxy, green, citrus, aldehydic and floral with a sweet, fatty, coconut nuance	0,75	0,00	0,00	0,00	1,01	0,00	0,67	0,79	0,00
7		2-Octenal, (E)	20,996	1058	Fatty	0,75	0,00	0,00	0,00	1,09	0,00	0,00	0,88	0,00
8		Nonanal	23,416	1103	Waxy, aldehydic, citrus, with a fresh slightly green lemon peel like nuance, and a cucumber fattiness	10,79	21,21	0,00	13,36	20,96	0,00	11,26	21,64	0,00
9		2-Nonenal, (E)	26,230	1158	Green, cucumber, aldehydic, fatty with a citrus nuance	2,62	2,70	0,00	2,33	2,85	0,00	2,12	0,00	0,00
10		2-Butenal, 2-methyl	6,945	780	Diffusive, pungent, green, ethareal, sharp penetrating, nutty and anisic	ND	ND	ND	ND	ND	ND	ND	ND	ND
11		Decanal	28,5	1203	Sweet, aldehydic, orange, waxy and citrus rind	5,79	5,48	0,00	6,97	5,65	0,00	5,61	4,69	0,00
12		Methional	12,77	903	Creamy tomato, potato skin and French fry, yeasty, bready, limburger cheese with a savory meaty brothy nuance	0,84	0,00	0,00	0,99	0,00	0,00	0,89	0,00	0,00
13		Benzeneacetaldehyde	20,172	1042	Green, honey	24,70	9,71	0,00	22,82	9,25	0,00	21,20	6,36	0,00
14		Benzaldehyde	15,705	960	Sharp, sweet, bitter, almond, cherry	5,91	5,55	0,00	5,41	4,21	0,00	5,01	5,25	0,00
15		Furfural	9,056	832	Sweet, woody, almond, bread baked	0,00	0,00	13,13	0,00	0,00	12,88	0,00	0,48	13,30
16		2-Butenal, 3-methyl	7,056	784	Sweet, fruity, green, nutty, cherry	ND	ND	ND	0,76	0,00	0,00	0,60	0,91	0,00
17		14-Heptadecenal	49,861	1867	-	ND	ND	ND	ND	ND	ND	ND	ND	ND
18		2-Dodecenal, (E)-	23,428	1103	-	ND	ND	ND	ND	ND	ND	ND	ND	ND
19		2-Propenal	19,246	1025	-	ND	ND	ND	ND	ND	ND	ND	ND	ND
20		Prenal	7,055	784	Sweet, fruity, pungent, brown and nutty with an almond and cherry nuance	ND	ND	ND	ND	ND	ND	ND	ND	ND
		TOTAL				61,33	51,29	13,13	61,29	53,86	12,88	55,41	53,21	13,30
	Alkanes													
21		Dodecane	28,221	1197	-	1,27	3,94	0,00	0,00	6,04	0,00	1,39	5,65	0,00
22		Tetradecane	37,398	1398	-	5,63	10,48	0,00	5,53	11,74	0,00	5,46	9,95	0,00
23		Hexadecane	41,601	1498	-	2,09	5,05	0,00	0,00	3,27	0,00	1,94	4,99	0,00
24		4,7-Dimethylundecane	41,591	1498	-	ND	ND	ND	ND	ND	ND	ND	ND	ND
25		3,3-Dimethylhexane	31,757	1272	-	ND	ND	ND	ND	ND	ND	ND	ND	ND
26		Phenethyl octanoate	34,364	1329	-	ND	ND	ND	ND	ND	ND	ND	ND	ND
27		2,2-Dimethylbutane	39,939	1458	-	ND	ND	ND	ND	ND	ND	ND	ND	ND
28		Decane	34,028	1321	-	ND	ND	ND	ND	ND	ND	ND	ND	ND
29		5-Octadecene, (E)	37,098	1391	-	ND	ND	ND	ND	ND	ND	ND	ND	ND
		TOTAL				8,99	19,47	0,00	5,53	21,05	0,00	8,79	20,59	0,00
	Ketones													
30		Acetophenone	21,325	1064	Sweet, pungent, hawthorn, mimosa, almond, acacia, chemical	0,24	0,00	0,24	0,00	0,00	0,00	0,00	0,48	0,38
31		1-Hydroxy-2-butanone	6,337	757	Coffee, sweet, brown, coffee, musty, grain- and pyrazine-like with malt and butterscotch nuances	0,00	0,00	0,00	1,04	0,00	0,00	1,13	0,00	0,00
32		1-Acetoxy-2-butanone	16,032	966	-	0,00	0,00	0,00	0,63	0,00	0,00	0,64	0,00	0,00
33		4-Hydroxy-3-hexanone	7,607	802	-	0,00	0,00	0,00	0,32	0,00	0,00	0,32	0,00	0,00
34		2,3-Pentanedione	4,194	678	Buttery, nutty, toasted, caramellic, diacetyl and acetoin notes	ND	ND	ND	ND	ND	ND	ND	ND	ND
35		Isobutyl ketone	16,25	970	-	ND	ND	ND	ND	ND	ND	ND	ND	ND
		TOTAL				0,24	0,00	0,24	1,99	0,00	0,00	2,09	0,48	0,38
	Phenol													
36		2-Methoxy-4-vinylphenol	33,528	1310	Sweet, spicy, clove carnation, phenolic, peppery, smoky, woody, powdery	0,00	0,00	10,74	0,00	0,00	10,02	0,00	0,00	0,00
		TOTAL				0,00	0,00	10,74	0,00	0,00	10,02	0,00	0,00	0,00
	Alcohois													
37		Isopentyl alcohol	5,199	715	Fermented, fusel, alcoholic, pungent, etherial, cognac, fruity, banana, molasses	0,00	1,21	0,00	1,72	0,45	0,00	0,00	0,71	0,00
38		Prenyl alcohol	6,667	770	Sweet, fruity, alcoholic with a green nuance	0,00	0,00	0,00	0,45	0,00	0,00	0,00	0,47	0,00
39		Furfuryl alcohol	10,558	862	Alcoholic chemical, musty, sweet, caramel, bread, coffee	0,00	0,00	35,48	0,00	0,00	34,16	0,00	0,00	39,62
40		1-Hexanol	10,945	870	Pungent, etherial, fusel oil, fruity and alcoholic, sweet with a green top note	0,00	2,23	0,00	ND	ND	ND	3,54	6,77	0,00
41		1-Octen-3-ol	16,843	981	Fresh, fungal, fruity, genuine, mushroom, earthy, violet, melon, humus	1,46	2,82	0,00	1,58	1,98	0,00	1,30	2,55	0,00
42		Benzyl alcohol	19,761	1035	Floral, rose, phenolic, balsamic	5,49	7,13	0,00	3,84	9,69	0,00	5,25	10,11	0,00
43		1-Octanol	21,754	1072	Waxy, green, orange, aldehydic, rose, mushroom	0,00	1,78	0,00	1,05	2,17	0,00	0,59	2,12	0,00
44		Phenylethyl Alcohol	23,811	1111	Floral, rose, dried rose	3,17	5,90	0,00	5,59	4,37	0,00	3,54	6,77	0,00
45		Maltol	23,847	1112	Sweet, caramellic, cotton, candy, jammy fruity, bread baked	0,00	0,00	3,90	0,00	0,00	3,96	0,00	0,00	4,14
46		Ethylhexanol	19,514	965	-	0,00	0,00	0,00	0,00	1,53	0,00	0,00	1,68	0,00
47		5-Methyl-2-furfuryl alcohol	15,494	956	-	ND	ND	ND	ND	ND	ND	ND	ND	ND
48		2-Hexenol	19,498	1030	Fresh fatty green, fruity, vegetative, with leafy and herbal nuances	ND	ND	ND	ND	ND	ND	0,51	0,00	0,00
49		1-Heptanol, 6-methyl	21,772	1072	-	ND	ND	ND	ND	ND	ND	ND	ND	ND
50		1-Dodecanol	44,651	1576	Earthy, soapy, waxy, fatty, honey, coconut	ND	ND	ND	ND	ND	ND	0,54	0,00	0,00
51		Z-2-Dodecenol	49,856	1768	-	ND	ND	ND	ND	ND	ND	0,00	0,72	0,00

52		3-Buten-2-ol	33,727	1314	-	ND	ND	ND	ND	ND	ND	0,31	0,18	0,00
53		Isoamyl alcohol	5,243	717	Fusel, alcoholic, pungent, etherial, cognac, fruity, banana and molasses	ND	ND	ND	ND	ND	ND	ND	ND	ND
54		2-Buten-1-ol, propanoate	7,44	798	-	ND	ND	ND	ND	ND	ND	ND	ND	ND
55		1-Heptanol	26,938	1172	-	ND	ND	ND	ND	ND	ND	ND	ND	ND
		TOTAL			-	10,13	21,07	39,38	14,23	20,19	38,12	15,59	32,08	43,77
	Ester													
56		Methyl salicylate	27,805	1189	Wintergreen, minty	0,91	1,13	0,00	ND	ND	ND	ND	ND	ND
57		Undecyl acetate	22,644	1088	Oily, clean, waxy	0,00	0,00	0,80	0,00	0,00	0,72	ND	ND	ND
58		Furfuryl acetate	17,505	993	Sweet fruity, banana, horseradish	0,00	0,00	1,58	0,00	0,00	1,65	0,00	0,00	1,87
59		Ethyl isovalerate	10,068	852	Sweet, diffusive, estry, fruity, sharp, pineapple, apple, green and orange	ND	ND	ND	ND	3,27	0,00	0,00	ND	ND
60		Ethyl phenacetate	30,212	1239	Floral honey, rosy with balsamic dark chocolate and cocoa notes with anisic black licorice nuances	ND	ND	ND	1,25	0,00	0,00	0,78	0,00	0,00
61		Propyl propionate	7,403	797	Sharp, chemical, pungent with sweet fruity lift notes	ND	ND	ND	ND	ND	ND	ND	ND	ND
62		Ethylmethylacetic acid	11,581	883	Etherial, fruity, sweet, grape and rum-like	1,30	0,00	0,00	0,00	0,00	0,00	1,28	0,00	0,00
63		5-Hexenyl propionate	19,474	1029	Green, fruity apple and pear pulp with creamy and powdery nuances	ND	ND	ND	ND	ND	ND	ND	ND	ND
64		Ethyl hexadecanoate	56,627	2200	Waxy, fruity, creamy and milky with a balsamic nuance	0,00	0,88	0,00	ND	ND	ND	0,62	0,00	0,00
65		Ethylhexanol	19,47	1029	-				ND	ND	ND			
		TOTAL				2,21	2,01	2,39	1,25	3,27	2,36	2,68	0,00	1,87
	Furan													
66		5-Hydroxymethylfurfural	29,866	1232	Fatty, buttery, musty, waxy, caramellic	0,00	0,00	5,96	0,00	0,00	5,45	ND	ND	ND
67		Furan, 2-pentyl	17,295	989	Fruity, green, earthy, beany, vegetable, metallic	ND	ND	ND	ND	ND	ND	ND	ND	ND
68		Furfural, 5-methy	15,82	962	Spicy, caramellic, maple	0,00	0,00	11,52	0,00	0,00	11,69	0,00	0,00	12,61
69		2- Acetylfuran	12,943	910	Sweet, balsamic, almond, cocoa, caramellic, coffee	0,00	0,00	1,38	0,00	0,00	1,37	0,00	0,00	1,68
70		Dihydro-2-methyl-3(2H)-furanone	7,878	808	Sweet and solvent-like with a brown, rummy and nut-like nuance	ND	ND	ND	ND	ND	ND	ND	ND	ND
71		2-Butylfuran	15,267	952	Fruity, winey, sweet, spicy	ND	ND	ND	ND	ND	ND	ND	ND	ND
72		2-Amylfuran	17,33	990	-	ND	ND	ND	ND	ND	ND	ND	ND	ND
		TOTAL				0,00	0,00	18,86	0,00	0,00	18,51	0,00	0,00	14,29
	Pyrazines													
73		Pyrazine, 3-ethyl-2,5-dimethyl	21,97	1076	Potato, cocoa, roasted, nutty	ND	ND	ND	ND	ND	ND	ND	ND	ND
74		2-Ethyl-6-methylpyrazine	17,795	998	Roasted potato	0,00	0,00	0,40	0,00	0,00	0,58	ND	ND	ND
75		2-Ethyl-3-methylpyrazine	18,009	1002	Nutty, peanut, musty, corn raw, earthy, bready	0,00	0,00	1,55	0,00	0,00	1,70	ND	ND	ND
76		Furfuryl formate	12,739	906	Ethereal	0,00	0,00	0,55	0,00	0,00	0,65	0,00	0,00	0,60
77		Methylpyrazine	8,648	823	Nutty, cocoa, roasted, chocolate, peanut green	0,00	0,00	5,07	0,00	0,00	4,01	0,00	0,00	5,41
78		Pyrazine	5,281	718	Sour, fishy, ammoniacal	0,00	0,00	0,57	0,00	0,00	0,55	0,00	0,00	0,68
79		2,5-Dimethylpyrazine	13,224	915	-	ND	ND	ND	ND	ND	ND	ND	ND	ND
		TOTAL				0,00	0,00	8,14	0,00	0,00	7,49	0,00	0,00	6,69
	Pyrrole													
80		2-Acetylpyrrole	21,62	1069	Musty, nut, skin, cherry maraschino, cherry coumarinic, licorice, bready, walnut bready	0,00	0,00	2,42	0,00	0,00	2,17	0,00	0,00	2,55
81		1- Ethylpyrrole	18,822	959	Burnt	ND	ND	ND	0,00	0,00	1,13	0,00	0,00	1,65
82		2 Formylpyrrole	19,045	961	Musty, beefy, coffee	ND	ND	ND	0,00	0,00	1,01	ND	ND	ND
83		1 Furfurylpyrrole	27,138	1152	Vegetative, careal, bready, radish, mushroom and potato nuances	ND	ND	ND	0,00	0,00	0,50	0,00	0,00	0,50
		TOTAL				0,00	0,00	2,42	0,00	0,00	4,81	0,00	0,00	4,70
	FFA/Acids													
84		Hexanoic acid	17,283	989	Sour, fatty, sweaty, cheesy	1,22	1,19	0,00	0,00	1,08	0,00	0,05	2,27	0,00
85		Senecioic acid	12,772	907	Milky, roasted, burnt	ND	ND	ND	ND	ND	ND	0,71	0,00	0,00
86		Butanoic acid, 2-methyl	11,426	880	Pungent acidic cheesy roquefort cheese cheesy	1,36	0,74	0,00	2,13	0,00	0,00	ND	ND	ND
87		Isovaleric acid	10,081	853	Sour, sweaty ,cheesy, tropical	14,50	3,44	0,71	12,30	3,83	0,65	15,30	1,86	0,78
88		Propanoic acid	4,856	703	Pungent acidic, cheesy, vinegar	0,00	0,00	0,77	0,00	0,00	0,89	0,00	0,00	1,32
89		3-Methylvaleric acid	10,06	852	Animalic, sharp acidic cheesey, green with a fruity sweaty nuance	ND	ND	ND	ND	ND	ND	2,74	0,00	0,00
90		n-Hexadecanoic acid	56,204	2173	-	ND	ND	ND	ND	ND	ND	ND	ND	ND
91		Butanoic acid	12,553	902	-	ND	ND	ND	ND	ND	ND	0,56	0,00	0,00
92		Nonanoic acid	31,756	1272	-	ND	ND	ND	ND	ND	ND	ND	ND	ND
		Total			-	17,08	5,37	1,48	14,43	4,91	1,54	19,37	4,13	2,10
	Pyridines													
93		Pyridine	5,689	733	Nutty	0,00	0,00	1,48	0,00	0,00	1,48	0,00	0,00	1,71
		TOTAL			-	0,00	0,00	1,48	0,00	0,00	1,48	0,00	0,00	1,71
	Lactones													
94		beta.-Angelica lactone	14,458	937	-	ND	ND	ND	ND	ND	ND	ND	ND	ND
95		Delta.-Octalactone	13,979	928	Sweet, coconut, creamy, coumarin and lactonic with a green, fatty nuance	ND	ND	ND	ND	ND	ND	ND	ND	ND
		TOTAL				0,00	0,00	0,00	0,00	0,00	0,00	0,00	0,00	0,00
	Other													
96		Betula oil	27,805	1189	Sweet, methyl salicylate, aromatic, spicy and herbal with a phenolic nuance	ND	ND	ND	ND	ND	ND	0,70	2,00	0,00
97		Corylon	19,34	1104	-	ND	ND	ND	ND	ND	ND	ND	ND	ND
98		Linalol	23,178	1100	-	ND	ND	ND	ND	ND	ND	ND	ND	ND
99		Furyl ethyl ketone	18,229	1006	-	ND	ND	ND	ND	ND	ND	ND	ND	ND
100		Isoamyl nitrite	31,709	1271	-	ND	ND	ND	ND	ND	ND	ND	ND	ND
		TOTAL				0,00	0,00	0,00	0,00	0,00	0,00	0,70	2,00	0,00

[1]ND means that the compound was not detected. All odors descriptor were find on http://www.thegoodscentscompany.com/.

Table S5. Volatile compounds (%) and sensory descriptors determined by HS-SPME/GC-MS in the MGS Ametista coffee variety (A) fermented with different inoculums[1].

No.	Class	Volatile compounds	Retention Time	Retention Index	Odor descriptors	N			CA11			B10		
	Aldehydes					48 hours	Dry	Roasted	48 hours	Dry	Roasted	48 hours	Dry	Roasted
1		3-Methylbutanal	3,47	651	Ethereal, aldehydic, chocolate, peach, fatty	7,60	0,00	0,00	8,16	2,15	0,00	7,09	0,90	0,00
2		2-Methylbutanal	3,610	657	Musty, cocoa, phenolic, coffee, nutty, malty, fermented, fatty alcoholic	1,91	1,93	0,00	2,64	2,10	0,00	2,58	0,75	0,00
3		Hexanal	7,542	801	Green, fatty, leafy, vegetative, fruity and clean with a woody nuance	0,97	4,24	0,00	2,04	4,74	0,00	1,90	3,61	0,00
4		Heptanal	12,519	902	Fresh, aldehydic fatty, green, herbal, cognac, ozone	0,00	0,44	0,00	0,00	0,53	0,00	0,00	0,36	0,00
5		2-Heptenal, (E)	15,536	957	Green	0,48	1,47	0,00	1,59	2,02	0,00	0,35	1,46	0,00
6		Octanal	18,044	1003	Waxy, green, citrus, aldehydic and floral with a sweet, fatty, coconut nuance	0,28	0,00	0,00	0,00	0,96	0,00	ND	ND	ND
7		2-Octenal, (E)	20,996	1058	Fatty	0,61	0,92	0,00	0,64	1,16	0,00	0,61	1,54	0,00
8		Nonanal	23,416	1103	Waxy, aldehydic, citrus, with a fresh slightly green lemon peel like nuance, and a cucumber fattiness	6,21	16,58	0,00	8,15	15,57	0,00	7,45	16,88	0,00
9		2-Nonenal, (E)	26,230	1158	Green, cucumber, aldehydic, fatty with a citrus nuance	1,72	1,76	0,00	1,48	2,34	0,00	1,87	2,33	0,00
10		2-Butenal, 2-methyl	6,945	780	Diffusive, pungent, green, ethareal, sharp penetrating, nutty and anisic	0,35	0,00	0,00	0,61	0,90	0,00			
11		Decanal	28,5	1203	Sweet, aldehydic, orange, waxy and citrus rind	2,40	4,64	0,00	3,50	3,72	0,00	2,78	4,32	0,00
12		Methional	12,77	903	Creamy tomato, potato skin and French fry, yeasty, bready, limburger cheese with a savory meaty brothy nuance	0,95	0,00	0,00	ND	ND	ND	ND	ND	ND
13		Benzeneacetaldehyde	20,172	1042	Green, honey	40,64	16,28	0,00	36,49	12,79	0,00	33,04	9,53	0,00
14		Benzaldehyde	15,705	960	Sharp, sweet, bitter, almond, cherry	7,86	8,64	0,00	8,33	7,25	0,00	6,67	5,66	0,00
15		Furfural	9,056	832	Sweet, woody, almond, bread baked	0,30	0,00	10,56	0,00	0,00	14,53	0,35	0,15	0,23
16		2-Butenal, 3-methyl	7,056	784	Sweet, fruity, green, nutty, cherry	ND	ND	ND	ND	ND	ND	0,59	0,66	0,00
17		14-Heptadecenal	49,861	1867	-	0,00	0,31	0,00	ND	ND	ND	0,35	0,00	0,00
18		2-Dodecenal, (E)-	23,428	1103	-	ND	ND	ND	ND	ND	ND	0,00	0,00	2,61
19		2-Propenal	19,246	1025	-	ND	ND	ND	ND	ND	ND	0,05	0,00	0,00
20		Prenal	7,055	784	Sweet, fruity, pungent, brown and nutty with an almond and cherry nuance	ND	ND	ND	ND	ND	ND			
		TOTAL				72,28	57,20	10,56	73,63	56,21	14,53	65,69	48,16	2,85
	Alkanes													
21		Dodecane	28,221	1197	-	0,78	3,34	0,00	1,09	4,42	0,00	1,20	4,07	0,83
22		Tetradecane	37,398	1398	-	3,70	7,27	0,00	3,47	1,60	0,00	3,85	7,49	0,00
23		Hexadecane	41,601	1498	-	1,12	1,44	0,00	0,00	0,12	0,00	1,65	1,45	0,00
24		4,7-Dimethylundecane	41,591	1498	-	ND	ND	ND	ND	ND	ND	0,68	0,00	0,00
25		3,3-Dimethylhexane	31,757	1272	-	ND	ND	ND	ND	ND	ND	2,62	0,00	0,00
26		Phenethyl octanoate	34,364	1329	-	ND	ND	ND	ND	ND	ND	0,34	0,00	0,00
27		2,2-Dimethylbutane	39,939	1458	-	ND	ND	ND	ND	ND	ND	0,31	0,00	0,00
28		Decane	34,028	1321	-	ND	ND	ND	ND	ND	ND	ND	ND	ND
29		5-Octadecene, (E)	37,098	1391	-	ND	ND	ND	ND	ND	ND	ND	ND	ND
		TOTAL				5,60	12,06	0,00	4,56	6,15	0,00	10,65	13,02	0,83
	Ketones													
30		Acetophenone	21,325	1064	Sweet, pungent, hawthorn, mimosa, almond, acacia, chemical	0,25	0,29	0,00	0,27	0,43	0,00	0,29	0,31	0,00
31		1-Hydroxy-2-butanone	6,337	757	Coffee, sweet, brown, coffee, musty, grain- and pyrazine-like with malt and butterscotch nuances	0,23	0,00	0,56	0,00	0,00	1,09	0,00	0,00	0,90
32		1-Acetoxy-2-butanone	16,032	966	-	0,00	0,00	8,48	0,00	0,00	0,59	0,00	0,00	1,54
33		4-Hydroxy-3-hexanone	7,607	802	-	0,00	0,00	0,12	0,00	0,00	0,32	0,00	0,00	0,68
34		2,3-Pentanedione	4,194	678	Buttery, nutty, toasted, caramellic, diacetyl and acetoin notes	ND	ND	ND	ND	ND	ND	0,00	0,00	0,87
35		Isobutyl ketone	16,25	970	-	ND	ND	ND	ND	ND	ND	0,42	0,00	0,00
		TOTAL				0,48	0,29	9,16	0,27	0,43	2,01	0,71	0,31	3,99
	Phenol													
36		2-Methoxy-4-vinylphenol	33,528	1310	Sweet, spicy, clove carnation, phenolic, peppery, smoky, woody, powdery	0,00	0,00	5,51	0,00	0,00	8,62	0,00	0,00	5,87
		TOTAL				0,00	0,00	5,51	0,00	0,00	8,62	0,00	0,00	5,87
	Alcohois													
37		Isopentyl alcohol	5,199	715	Fermented, fusel, alcoholic, pungent, etherial, cognac, fruity, banana, molasses	ND	ND	ND	0,40	1,30	0,00	0,04	1,21	0,00
38		Prenyl alcohol	6,667	770	Sweet, fruity, alcoholic with a green nuance	0,23	1,23	0,00	0,39	0,52	0,00	ND	ND	ND
39		Furfuryl alcohol	10,558	862	Alcoholic chemical, musty, sweet, caramel, bread, coffee	6,30	0,00	28,16	0,00	0,00	34,83	0,00	0,00	23,90
40		1-Hexanol	10,945	870	Pungent, etherial, fusel oil, fruity and alcoholic, sweet with a green top note	ND	ND	ND	ND	ND	ND	0,00	2,78	0,00
41		1-Octen-3-ol	16,843	981	Fresh, fungal, fruity, genuine, mushroom, earthy, violet, melon, humus	1,16	1,81	0,00	1,62	2,52	0,00	1,41	2,65	0,00
42		Benzyl alcohol	19,761	1035	Floral, rose, phenolic, balsamic	6,17	10,13	0,00	4,80	13,83	0,00	0,00	1,11	0,00
43		1-Octanol	21,754	1072	Waxy, green, orange, aldehydic, rose, mushroom	0,00	1,22	0,00	0,00	1,13	0,00	0,51	1,53	0,00

44		Phenylethyl Alcohol	23,811	1111	Floral, rose, dried rose	4,54	11,05	0,00	6,78	10,08	0,00	5,02	12,36	0,00
45		Maltol	23,847	1112	Sweet, caramellic, cotton, candy, jammy fruity, bread baked	0,00	0,00	2,19	0,00	0,00	3,61	0,00	0,00	3,08
46		Ethylhexanol	19,514	965	-	ND	ND	ND	ND	ND	ND	ND	ND	ND
47		5-Methyl-2-furfuryl alcohol	15,494	956	-	0,00	0,00	0,30	ND	ND	ND	0,00	0,00	8,11
48		2-Hexenol	19,498	1030	Fresh fatty green, fruity, vegetative, with leafy and herbal nuances	ND	ND	ND	0,00	1,15	0,00	ND	ND	ND
49		1-Heptanol, 6-methyl	21,772	1072	-	ND	ND	ND	ND	ND	ND	0,38	0,00	0,00
50		1-Dodecanol	44,651	1576	Earthy, soapy, waxy, fatty, honey, coconut	ND	ND	ND	ND	ND	ND	ND	ND	ND
51		Z-2-Dodecenol	49,856	1768	-	ND	ND	ND	ND	ND	ND	0,00	0,24	0,00
52		3-Buten-2-ol	33,727	1314	-	ND	ND	ND	0,00	1,60	0,00	0,14	0,00	0,00
53		Isoamyl alcohol	5,243	717	Fusel, alcoholic, pungent, etherial, cognac, fruity, banana and molasses	0,00	1,23	0,00	ND	ND	ND			
54		2-Buten-1-ol, propanoate	7,44	798	-	ND	ND	ND	ND	ND	ND	0,28	0,53	0,00
55		1-Heptanol	26,938	1172	-	ND	ND	ND	ND	ND	ND	0,00	0,41	0,00
		TOTAL			-	18,40	26,67	30,65	13,99	32,13	38,44	7,77	22,82	35,09
	Ester													
56		Methyl salicylate	27,805	1189	Wintergreen, minty	0,66	0,00	0,00	0,54	0,67	0,00	ND	ND	ND
57		Undecyl acetate	22,644	1088	Oily, clean, waxy	ND	ND	ND	0,00	0,00	0,66	ND	ND	ND
58		Furfuryl acetate	17,505	993	Sweet fruity, banana, horseradish	0,00	0,00	1,14	0,00	0,00	1,53	0,00	0,00	1,50
59		Ethyl isovalerate	10,068	852	Sweet, diffusive, estry, fruity, sharp, pineapple, apple, green and orange	ND	ND	ND	ND	ND	ND	ND	ND	ND
60		Ethyl phenacetate	30,212	1239	Floral honey, rosy with balsamic dark chocolate and cocoa notes with anisic black licorice nuances	0,38	0,00	0,00	1,03	0,00	0,00	0,97	0,00	0,00
61		Propyl propionate	7,403	797	Sharp, chemical, pungent with sweet fruity lift notes	0,63	0,00	0,00	ND	ND	ND	ND	ND	ND
62		Ethylmethylacetic acid	11,581	883	Etherial, fruity, sweet, grape and rum-like	ND	ND	ND	ND	ND	ND	1,24	0,00	0,00
63		5-Hexenyl propionate	19,474	1029	Green, fruity apple and pear pulp with creamy and powdery nuances	ND	ND	ND	ND	ND	ND	0,34	0,00	0,00
64		Ethyl hexadecanoate	56,627	2200	Waxy, fruity, creamy and milky with a balsamic nuance	ND	ND	ND	ND	ND	ND	ND	ND	ND
65		Ethylhexanol	19,47	1029	-	ND	ND	ND	ND	ND	ND	ND	ND	ND
		TOTAL				1,67	0,00	1,14	1,57	0,67	2,19	2,55	0,00	1,50
	Furan													
66		5-Hydroxymethylfurfural	29,866	1232	Fatty, buttery, musty, waxy, caramellic	0,00	0,00	2,67	0,00	0,00	4,43	0,00	0,00	8,92
67		Furan, 2-pentyl	17,295	989	Fruity, green, earthy, beany, vegetable, metallic	0,70	0,00	0,00	ND	ND	ND	ND	ND	ND
68		Furfural, 5-methy	15,82	962	Spicy, caramellic, maple	0,00	0,00	0,29	ND	ND	ND	0,00	0,00	8,28
69		2- Acetylfuran	12,943	910	Sweet, balsamic, almond, cocoa, caramellic, coffee	ND	ND	ND	0,00	0,00	1,47	0,00	0,00	1,64
70		Dihydro-2-methyl-3(2H)-furanone	7,878	808	Sweet and solvent-like with a brown, rummy and nut-like nuance	ND	ND	ND	ND	ND	ND	0,00	0,00	3,24
71		2-Butylfuran	15,267	952	Fruity, winey, sweet, spicy	ND	ND	ND	ND	ND	ND	0,00	0,00	0,31
72		2-Amylfuran	17,33	990	-	ND	ND	ND	ND	ND	ND	0,98	0,00	0,00
		TOTAL				0,70	0,00	2,96	0,00	0,00	5,90	0,98	0,00	22,39
	Pyrazines													
73		Pyrazine, 3-ethyl-2,5-dimethyl	21,97	1076	Potato, cocoa, roasted, nutty	0,00	0,00	1,07	ND	ND	ND	ND	ND	ND
74		2-Ethyl-6-methylpyrazine	17,795	998	Roasted potato	0,00	0,00	0,54	0,00	0,00	1,67	0,00	0,00	1,73
75		2-Ethyl-3-methylpyrazine	18,009	1002	Nutty, peanut, musty, corn raw, earthy, bready	0,00	0,00	1,53	0,00	0,00	0,46	0,00	0,00	1,39
76		Furfuryl formate	12,739	906	Ethereal	0,00	0,00	0,61	0,00	0,00	0,61	0,00	0,00	1,35
77		Methylpyrazine	8,648	823	Nutty, cocoa, roasted, chocolate, peanut green	0,00	0,00	3,09	0,00	0,00	5,20	0,00	0,00	11,96
78		Pyrazine	5,281	718	Sour, fishy, ammoniacal	0,00	0,00	0,27	0,00	0,00	0,64	0,00	0,00	1,38
79		2,5-Dimethylpyrazine	13,224	915	-	ND	ND	ND	ND	ND	ND	0,00	0,00	0,89
		TOTAL				0,00	0,00	7,11	0,00	0,00	8,57	0,00	0,00	18,70
	Pyrrole													
80		2-Acetylpyrrole	21,62	1069	Musty, nut, skin, cherry maraschino, cherry coumarinic, licorice, bready, walnut bready	0,00	0,00	1,44	0,00	0,00	2,05	0,00	0,00	1,79
81		1- Ethylpyrrole	18,822	959	Burnt	ND	ND	ND	0,00	0,00	1,24	ND	ND	ND
82		2 Formylpyrrole	19,045	961	Musty, beefy, coffee	ND	ND	ND	0,00	0,00	1,18	ND	ND	ND
83		1 Furfurylpyrrole	27,138	1152	Vegetative, careal, bready, radish, mushroom and potato nuances	0,00	0,00	0,41	0,00	0,00	0,49	ND	ND	ND
		TOTAL				0,00	0,00	1,85	0,00	0,00	4,97	0,00	0,00	1,79
	FFA/Acids													
84		Hexanoic acid	17,283	989	Sour, fatty, sweaty, cheesy	0,47	1,81	0,00	0,00	0,93	0,00	0,00	1,93	0,00
85		Senecioic acid	12,772	907	Milky, roasted, burnt	ND	ND	ND	ND	ND	ND	1,59	0,00	0,00
86		Butanoic acid, 2-methyl	11,426	880	Pungent acidic cheesy roquefort cheese cheesy	ND	ND	ND	1,11	0,00	0,00	0,00	0,00	0,47
87		Isovaleric acid	10,081	853	Sour, sweaty ,cheesy, tropical	6,36	0,70	28,16	2,56	0,00	0,88	3,62	0,40	0,69
88		Propanoic acid	4,856	703	Pungent acidic, cheesy, vinegar	0,00	0,00	0,54	0,00	0,00	1,00	0,00	0,00	1,03
89		3-Methylvaleric acid	10,06	852	Animalic, sharp acidic cheesey, green with a fruity sweaty nuance	ND	ND	ND	ND	ND	ND	ND	ND	ND
90		n-Hexadecanoic acid	56,204	2173	-	ND	ND	ND	ND	ND	ND	0,00	0,00	0,75
91		Butanoic acid	12,553	902	-	ND	ND	ND	ND	ND	ND	0,13	0,00	0,00
92		Nonanoic acid	31,756	1272	-	ND	ND	ND	0,00	2,95	0,00	ND	ND	ND
		Total			-	6,83	2,51	28,70	5,08	4,41	1,88	5,66	2,33	2,94
	Pyridines													
93		Pyridine	5,689	733	Nutty	0,00	0,00	0,78	0,00	0,00	1,57	0,00	0,00	1,76
		TOTAL			-	0,00	0,00	0,78	0,00	0,00	1,57	0,00	0,00	1,76
	Lactones													
94		beta.-Angelica lactone	14,458	937	-	ND	ND	ND	ND	ND	ND	0,00	0,00	0,16
95		Delta.-Octalactone	13,979	928	Sweet, coconut, creamy, coumarin and lactonic with a green, fatty nuance	ND	ND	ND	ND	ND	ND	0,00	0,00	0,27

		TOTAL				0,00	0,00	0,00	0,00	0,00	0,00	0,00	0,00	0,43
	Other													
96		Betula oil	27,805	1189	Sweet, methyl salicylate, aromatic, spicy and herbal with a phenolic nuance	ND	ND	ND	ND	ND	ND	0,42	1,18	0,00
97		Corylon	19,34	1104	-	0,00	0,00	0,97	ND	ND	ND	ND	ND	ND
98		Linalol	23,178	1100	-	0,56	0,00	0,00	ND	ND	ND	ND	ND	ND
99		Furyl ethyl ketone	18,229	1006	-	ND	ND	ND	ND	ND	ND	0,00	0,00	1,66
100		Isoamyl nitrite	31,709	1271	-	ND	ND	ND	ND	ND	ND	0,07	0,47	0,00
		TOTAL				0,56	0,00	0,97	0,00	0,00	0,00	0,49	1,64	1,66

[1]ND means that the compound was not detected. All odors descriptor were find on http://www.thegoodscentscompany.com/.

Table S6. Volatile compounds (%) and sensory descriptors determined by HS-SPME/GC-MS in the coffee variety MGS Catuaí Pioneira (CP) fermented with different inoculums[1].

No.	Class	Volatile compounds	Retention Time	Retention Index	Odor descriptors	N			CA11			B10		
	Aldehydes					48 hours	Dry	Roasted	48 hours	Dry	Roasted	48 hours	Dry	Roasted
1		3-Methylbutanal	3,47	651	Ethereal, aldehydic, chocolate, peach, fatty	9,22	0,90	0,00	7,50	1,35	0,00	8,08	0,78	0,00
2		2-Methylbutanal	3,610	657	Musty, cocoa, phenolic, coffee, nutty, malty, fermented, fatty alcoholic	2,03	0,50	0,00	1,15	0,84	0,00	1,71	0,69	0,00
3		Hexanal	7,542	801	Green, fatty, leafy, vegetative, fruity and clean with a woody nuance	0,93	5,02	0,00	2,19	6,05	0,00	2,38	3,05	0,00
4		Heptanal	12,519	902	Fresh, aldehydic fatty, green, herbal, cognac, ozone	ND	ND	ND	0,00	0,43	0,00	0,00	0,26	0,00
5		2-Heptenal, (E)	15,536	957	Green	ND	ND	ND	0,00	1,80	0,00	0,39	1,08	0,00
6		Octanal	18,044	1003	Waxy, green, citrus, aldehydic and floral with a sweet, fatty, coconut nuance	0,59	0,81	0,00	0,55	0,00	0,00	0,60	1,16	0,00
7		2-Octenal, (E)	20,996	1058	Fatty	0,85	1,09	0,00	0,80	1,75	0,00	0,98	0,89	0,00
8		Nonanal	23,416	1103	Waxy, aldehydic, citrus, with a fresh slightly green lemon peel like nuance, and a cucumber fattiness	8,33	15,47	0,00	8,67	19,34	0,00	9,60	19,30	0,00
9		2-Nonenal, (E)	26,230	1158	Green, cucumber, aldehydic, fatty with a citrus nuance	1,98	1,90	0,00	1,88	2,80	0,00	1,98	1,71	0,00
10		2-Butenal, 2-methyl	6,945	780	Diffusive, pungent, green, ethareal, sharp penetrating, nutty and anisic	ND	ND	ND	ND	ND	ND	ND	ND	ND
11		Decanal	28,5	1203	Sweet, aldehydic, orange, waxy and citrus rind	4,68	4,44	0,00	5,04	4,63	0,00	1,85	6,42	0,00
12		Methional	12,77	903	Creamy tomato, potato skin and French fry, yeasty, bready, limburger cheese with a savory meaty brothy nuance									
13		Benzeneacetaldehyde	20,172	1042	Green, honey	33,54	17,71	0,00	37,43	9,54	0,00	27,78	7,11	0,00
14		Benzaldehyde	15,705	960	Sharp, sweet, bitter, almond, cherry	7,06	2,33	0,00	7,23	4,66	0,00	6,58	5,16	0,00
15		Furfural	9,056	832	Sweet, woody, almond, bread baked	0,00	0,00	13,67	0,00	0,00	13,35	0,45	0,00	14,64
16		2-Butenal, 3-methyl	7,056	784	Sweet, fruity, green, nutty, cherry	0,61	0,66	0,00	0,00	1,26	0,00	ND	ND	ND
17		14-Heptadecenal	49,861	1867	-	ND	ND	ND	ND	ND	ND	ND	ND	ND
18		2-Dodecenal, (E)-	23,428	1103	-	ND	ND	ND	ND	ND	ND	ND	ND	ND
19		2-Propenal	19,246	1025	-	ND	ND	ND	ND	ND	ND	ND	ND	ND
20		Prenal	7,055	784	Sweet, fruity, pungent, brown and nutty with an almond and cherry nuance	ND	ND	ND	ND	ND	ND	0,64	0,00	0,00
		TOTAL				69,82	50,83	13,67	72,44	54,46	13,35	63,02	47,61	14,64
	Alkanes													
21		Dodecane	28,221	1197	-	0,89	2,83	0,00	1,01	4,94	0,00	1,85	6,42	1,85
22		Tetradecane	37,398	1398	-	4,11	6,88	0,00	4,20	9,68	0,00	4,63	9,21	4,63
23		Hexadecane	41,601	1498	-	1,67	3,17	0,00	2,03	2,94	0,00	0,00	4,98	0,00
24		4,7-Dimethylundecane	41,591	1498	-	ND	ND	ND	ND	ND	ND	ND	ND	ND
25		3,3-Dimethylhexane	31,757	1272	-	ND	ND	ND	ND	ND	ND	ND	ND	ND
26		Phenethyl octanoate	34,364	1329	-	ND	ND	ND	ND	ND	ND	ND	ND	ND
27		2,2-Dimethylbutane	39,939	1458	-	ND	ND	ND	ND	ND	ND	ND	ND	ND
28		Decane	34,028	1321	-	ND	ND	ND	ND	ND	ND	0,00	1,33	0,00
29		5-Octadecene, (E)	37,098	1391	-	ND	ND	ND	ND	ND	ND	0,00	1,15	0,00
		TOTAL				6,67	12,88	0,00	7,24	17,56	0,00	6,48	23,11	6,48
	Ketones													
30		Acetophenone	21,325	1064	Sweet, pungent, hawthorn, mimosa, almond, acacia, chemical	0,29	0,32	0,00	0,00	0,50	0,00	0,34	0,51	0,00
31		1-Hydroxy-2-butanone	6,337	757	Coffee, sweet, brown, coffee, musty, grain- and pyrazine-like with malt and butterscotch nuances	0,00	0,00	1,09	0,00	0,00	1,01	0,00	0,00	1,08
32		1-Acetoxy-2-butanone	16,032	966	-	0,00	0,00	0,77	0,00	0,00	0,67	ND	ND	ND
33		4-Hydroxy-3-hexanone	7,607	802	-	0,00	0,00	0,31	0,00	0,00	0,29	ND	ND	ND
34		2,3-Pentanedione	4,194	678	Buttery, nutty, toasted, caramellic, diacetyl and acetoin notes	ND	ND	ND	ND	ND	ND	ND	ND	ND
35		Isobutyl ketone	16,25	970	-	ND	ND	ND	ND	ND	ND	ND	ND	ND
		TOTAL				0,29	0,32	2,17	0,00	0,50	1,98	0,34	0,51	1,08
	Phenol													
36		2-Methoxy-4-vinylphenol	33,528	1310	Sweet, spicy, clove carnation, phenolic, peppery, smoky, woody, powdery	0,00	0,00	9,92	0,00	0,00	9,85	0,00	0,00	10,09
		TOTAL				0,00	0,00	9,92	0,00	0,00	9,85	0,00	0,00	10,09
	Alcohois													
37		Isopentyl alcohol	5,199	715	Fermented, fusel, alcoholic, pungent, etherial, cognac, fruity, banana, molasses	0,00	0,35	0,00	0,95	0,57	0,00	ND	ND	ND
38		Prenyl alcohol	6,667	770	Sweet, fruity, alcoholic with a green nuance	0,39	0,97	0,00	0,00	0,57	0,00	0,40	0,57	0,00
39		Furfuryl alcohol	10,558	862	Alcoholic chemical, musty, sweet, caramel, bread, coffee	0,00	0,00	35,96	0,00	0,00	35,48	0,00	0,00	37,62
40		1-Hexanol	10,945	870	Pungent, etherial, fusel oil, fruity and alcoholic, sweet with a green top note	0,00	1,73	0,00	ND	ND	ND	0,00	2,16	0,00
41		1-Octen-3-ol	16,843	981	Fresh, fungal, fruity, genuine, mushroom, earthy, violet, melon, humus	1,41	8,27	0,00	1,57	2,65	0,00	1,69	2,16	0,00
42		Benzyl alcohol	19,761	1035	Floral, rose, phenolic, balsamic	6,44	7,30	0,00	4,76	9,80	0,00	5,82	10,28	0,00
43		1-Octanol	21,754	1072	Waxy, green, orange, aldehydic, rose, mushroom	0,00	1,03	0,00	0,00	1,35	0,00	0,00	1,41	0,00
44		Phenylethyl Alcohol	23,811	1111	Floral, rose, dried rose	2,96	7,48	0,00	6,67	5,48	0,00	5,50	8,99	0,00
45		Maltol	23,847	1112	Sweet, caramellic, cotton, candy, jammy fruity, bread baked	0,00	0,00	3,49	0,00	0,00	3,77	0,00	0,00	3,88
46		Ethylhexanol	19,514	965	-	ND	ND	ND	ND	ND	ND	0,00	1,18	0,00
47		5-Methyl-2-furfuryl alcohol	15,494	956	-	ND	ND	ND	ND	ND	ND	ND	ND	ND
48		2-Hexenol	19,498	1030	Fresh fatty green, fruity, vegetative, with leafy and herbal nuances	ND	ND	ND	ND	ND	ND	ND	ND	ND

49		1-Heptanol, 6-methyl	21,772	1072	-	ND	ND	ND	ND	ND	ND	ND	ND	ND
50		1-Dodecanol	44,651	1576	Earthy, soapy, waxy, fatty, honey, coconut	ND	ND	ND	ND	ND	ND	ND	ND	ND
51		Z-2-Dodecenol	49,856	1768	-	ND	ND	ND	ND	ND	ND	0,48	0,00	0,00
52		3-Buten-2-ol	33,727	1314	-	ND	ND	ND	ND	ND	ND	0,31	0,00	0,00
53		Isoamyl alcohol	5,243	717	Fusel, alcoholic, pungent, etherial, cognac, fruity, banana and molasses	ND	ND	ND	ND	ND	ND	ND	ND	ND
54		2-Buten-1-ol, propanoate	7,44	798	-	ND	ND	ND	ND	ND	ND	ND	ND	ND
55		1-Heptanol	26,938	1172	-	ND	ND	ND	ND	ND	ND	ND	ND	ND
		TOTAL			-	11,20	27,13	39,45	13,95	20,41	39,25	14,19	26,75	41,50
	Ester													
56		Methyl salicylate	27,805	1189	Wintergreen, minty	0,58	0,52	0,00	0,00	0,88	0,00	ND	ND	ND
57		Undecyl acetate	22,644	1088	Oily, clean, waxy	0,00	0,00	0,56	0,00	0,00	0,71	ND	ND	ND
58		Furfuryl acetate	17,505	993	Sweet fruity, banana, horseradish	0,00	0,00	1,70	0,00	0,00	1,45	0,00	0,00	1,42
59		Ethyl isovalerate	10,068	852	Sweet, diffusive, estry, fruity, sharp, pineapple, apple, green and orange	ND	ND	ND	ND	ND	ND	ND	ND	ND
60		Ethyl phenacetate	30,212	1239	Floral honey, rosy with balsamic dark chocolate and cocoa notes with anisic black licorice nuances	ND	ND	ND	1,20	0,00	0,00	1,09	0,00	0,00
61		Propyl propionate	7,403	797	Sharp, chemical, pungent with sweet fruity lift notes	ND	ND	ND	ND	ND	ND	ND	ND	ND
62		Ethylmethylacetic acid	11,581	883	Etherial, fruity, sweet, grape and rum-like	1,49	0,00	0,00	1,36	0,00	0,00	1,95	0,00	0,00
63		5-Hexenyl propionate	19,474	1029	Green, fruity apple and pear pulp with creamy and powdery nuances	ND	ND	ND	ND	ND	ND	ND	ND	ND
64		Ethyl hexadecanoate	56,627	2200	Waxy, fruity, creamy and milky with a balsamic nuance	ND	ND	ND	ND	ND	ND	ND	ND	ND
65		Ethylhexanol	19,47	1029	-	0,00	0,92	0,00	ND	ND	ND	ND	ND	ND
		TOTAL				2,07	1,44	2,25	2,55	0,88	2,16	3,04	0,00	1,42
	Furan													
66		5-Hydroxymethylfurfural	29,866	1232	Fatty, buttery, musty, waxy, caramellic	0,00	0,00	5,14	0,00	0,00	4,45	0,00	0,00	5,16
67		Furan, 2-pentyl	17,295	989	Fruity, green, earthy, beany, vegetable, metallic	1,00	0,00	0,00	ND	ND	ND	ND	ND	ND
68		Furfural, 5-methy	15,82	962	Spicy, caramellic, maple	0,00	0,00	11,92	0,00	0,00	11,01	0,00	0,00	11,08
69		2- Acetylfuran	12,943	910	Sweet, balsamic, almond, cocoa, caramellic, coffee	0,00	0,00	1,74	0,00	0,00	1,26			
70		Dihydro-2-methyl-3(2H)-furanone	7,878	808	Sweet and solvent-like with a brown, rummy and nut-like nuance	ND	ND	ND	ND	ND	ND	ND	ND	ND
71		2-Butylfuran	15,267	952	Fruity, winey, sweet, spicy	ND	ND	ND	ND	ND	ND	ND	ND	ND
72		2-Amylfuran	17,33	990	-	0,00	3,16	0,00	1,26	0,00	0,00	1,46	0,00	0,00
		TOTAL				1,00	3,16	18,80	1,26	0,00	16,73	1,46	0,00	16,24
	Pyrazines													
73		Pyrazine, 3-ethyl-2,5-dimethyl	21,97	1076	Potato, cocoa, roasted, nutty	ND	ND	ND	ND	ND	ND	ND	ND	ND
74		2-Ethyl-6-methylpyrazine	17,795	998	Roasted potato	0,00	0,00	0,41	0,00	0,00	0,59	0,00	0,00	0,77
75		2-Ethyl-3-methylpyrazine	18,009	1002	Nutty, peanut, musty, corn raw, earthy, bready	0,00	0,00	1,70	0,00	0,00	1,62			
76		Furfuryl formate	12,739	906	Ethereal	0,00	0,00	0,61	0,00	0,00	0,55	0,00	0,00	0,54
77		Methylpyrazine	8,648	823	Nutty, cocoa, roasted, chocolate, peanut green	0,00	0,00	4,63	0,00	0,00	4,19	0,00	0,00	6,53
78		Pyrazine	5,281	718	Sour, fishy, ammoniacal	0,00	0,00	0,53	0,00	0,00	0,47	0,00	0,00	0,85
79		2,5-Dimethylpyrazine	13,224	915	-	ND	ND	ND	ND	ND	ND	ND	ND	ND
		TOTAL				0,00	0,00	7,88	0,00	0,00	7,42	0,00	0,00	8,70
	Pyrrole													
80		2-Acetylpyrrole	21,62	1069	Musty, nut, skin, cherry maraschino, cherry coumarinic, licorice, bready, walnut bready	0,00	0,00	2,16	0,00	0,00	2,23	0,00	0,00	2,40
81		1- Ethylpyrrole	18,822	959	Burnt	ND	ND	ND	0,00	0,00	1,09	ND	ND	ND
82		2 Formylpyrrole	19,045	961	Musty, beefy, coffee	ND	ND	ND	0,00	0,00	1,07	ND	ND	ND
83		1 Furfurylpyrrole	27,138	1152	Vegetative, careal, bready, radish, mushroom and potato nuances	ND	ND	ND	0,00	0,00	0,65	ND	ND	ND
		TOTAL				0,00	0,00	2,16	0,00	0,00	5,05	0,00	0,00	2,40
	FFA/Acids													
84		Hexanoic acid	17,283	989	Sour, fatty, sweaty, cheesy	0,20	3,30	0,00	0,00	1,90	0,00	0,00	1,65	0,00
85		Senecioic acid	12,772	907	Milky, roasted, burnt	1,60	0,00	0,00	ND	ND	ND	1,05	0,00	0,00
86		Butanoic acid, 2-methyl	11,426	880	Pungent acidic cheesy roquefort cheese cheesy	ND	ND	ND	ND	ND	ND	ND	ND	ND
87		Isovaleric acid	10,081	853	Sour, sweaty ,cheesy, tropical	7,15	0,93	1,06	2,43	2,38	0,61	6,04	0,00	1,44
88		Propanoic acid	4,856	703	Pungent acidic, cheesy, vinegar	0,00	0,00	1,17	0,00	0,00	0,76	0,00	0,00	0,72
89		3-Methylvaleric acid	10,06	852	Animalic, sharp acidic cheesey, green with a fruity sweaty nuance	ND	ND	ND	ND	ND	ND	ND	ND	ND
90		n-Hexadecanoic acid	56,204	2173	-	ND	ND	ND	0,00	0,00	1,52	ND	ND	ND
91		Butanoic acid	12,553	902	-	ND	ND	ND	ND	ND	ND	ND	ND	ND
92		Nonanoic acid	31,756	1272	-	ND	ND	ND	ND	ND	ND	ND	ND	ND
		Total				8,95	4,23	2,23	2,43	4,28	2,89	7,08	1,65	2,17
	Pyridines													
93		Pyridine	5,689	733	Nutty	0,00	0,00	1,47	0,00	0,00	1,31	0,00	0,00	1,38
		TOTAL			-	0,00	0,00	1,47	0,00	0,00	1,31	0,00	0,00	1,38
	Lactones				-									
94		beta.-Angelica lactone	14,458	937	-	ND	ND	ND	ND	ND	ND	ND	ND	ND
95		Delta.-Octalactone	13,979	928	Sweet, coconut, creamy, coumarin and lactonic with a green, fatty nuance	ND	ND	ND	ND	ND	ND	ND	ND	ND
		TOTAL				0,00	0,00	0,00	0,00	0,00	0,00	0,00	0,00	0,00
	Other													
96		Betula oil	27,805	1189	Sweet, methyl salicylate, aromatic, spicy and herbal with a phenolic nuance	ND	ND	ND	ND	ND	ND	0,87	1,30	0,00
97		Corylon	19,34	1104	-	ND	ND	ND	ND	ND	ND	ND	ND	ND
98		Linalol	23,178	1100	-	ND	ND	ND	ND	ND	ND	1,08	0,00	0,00
99		Furyl ethyl ketone	18,229	1006	-	ND	ND	ND	ND	ND	ND	ND	ND	ND
100		Isoamyl nitrite	31,709	1271	-	ND	ND	ND	ND	ND	ND	ND	ND	ND

TOTAL	0,00	0,00	0,00	0,00	0,00	0,00	1,95	1,30	0,00

[1]ND means that the compound was not detected. All odors descriptor were find on http://www.thegoodscentscompany.com/.

Table S7. Sensory evaluation notes on coffee drinks from genetic varieties of arabica coffee subjected to fermentation.

Parameters	N				CA11				B10			
	P2	A	CP	CA62	P2	A	CP	CA62	P2	A	CP	CA62
Fragrance/Aroma	7.72^{aA}	7.50^{aB}	7.50^{aA}	7.61^{aA}	7.78^{aA}	7.78^{aA}	7.50^{bA}	7.50^{bA}	7.61^{aA}	7.50^{aB}	7.61^{aA}	7.50^{aA}
Uniformity	10.00^{aA}	10.00^{aA}	10.00^{aA}	10.00^{aA}	10.00^{aA}	10.00^{aA}	10.00^{aA}	10.00^{aA}	10.00^{aA}	10.00^{aA}	10.00^{aA}	10.00^{aA}
Clean Cup	10.00^{aA}	10.00^{aA}	10.00^{aA}	10.00^{aA}	10.00^{aA}	10.00^{aA}	10.00^{aA}	10.00^{aA}	10.00^{aA}	10.00^{aA}	10.00^{aA}	10.00^{aA}
Sweetness	10.00^{aA}	10.00^{aA}	10.00^{aA}	10.00^{aA}	10.00^{aA}	10.00^{aA}	10.00^{aA}	10.00^{aA}	10.00^{aA}	10.00^{aA}	10.00^{aA}	10.00^{aA}
Flavor	7.94^{aA}	7.67^{aA}	7.83^{aA}	7.83^{aA}	8.06^{aA}	7.78^{aA}	7.78^{aA}	7.83^{aA}	7.94^{aA}	7.83^{aA}	7.89^{aA}	7.78^{aA}
Acidity	7.89^{aA}	7.50^{bA}	7.67^{bA}	7.67^{bA}	7.94^{aA}	7.61^{aA}	7.78^{aA}	7.67^{aA}	7.78^{aA}	7.61^{aA}	7.67^{aA}	7.56^{aA}
Body	7.94^{aA}	7.83^{aA}	7.94^{aA}	8.00^{aA}	8.00^{aA}	7.89^{aA}	7.94^{aA}	7.89^{aA}	8.00^{aA}	8.00^{aA}	7.89^{aA}	7.94^{aA}
Aftertaste	7.72^{aA}	7.50^{aA}	7.50^{aA}	7.72^{aA}	7.94^{aA}	7.56^{aA}	7.67^{aA}	7.61^{aA}	7.89^{aA}	7.67^{aA}	7.78^{aA}	7.56^{aA}
Balance	7.56^{aB}	7.50^{aA}	7.50^{aA}	7.50^{aA}	7.78^{aA}	7.50^{bA}	7.50^{bA}	7.61^{bA}	7.72^{aA}	7.61^{aA}	7.56^{aA}	7.50^{aA}
Overall	7.61^{aA}	7.50^{aA}	7.50^{aA}	7.67^{aA}	7.78^{aA}	7.50^{aA}	7.61^{aA}	7.50^{aA}	7.50^{aA}	7.50^{aA}	7.61^{aA}	7.50^{aA}
Final score	84.39^{aA}	83.00^{aA}	83.44^{aA}	84.00^{aA}	85.28^{aA}	83.61^{bA}	83.78^{bA}	83.61^{bA}	84.44^{aA}	83.72^{aA}	84.00^{aA}	83.33^{bA}

[1] Mean followed by lower case letters and upper case letters on the same line show statistical difference by the Scott-Knott test. ($p<0.05$).

Figure S3. Pearson correlation of different arabica coffee cultivars fermented by yeast starters (A: CA62; B: A; C: P2; D: CP) - (*) Correlation is significant at a 0.05 level; (**) Correlation is significant at a 0.01 level; (***) Correlation is significant at a 0.001 level.

A

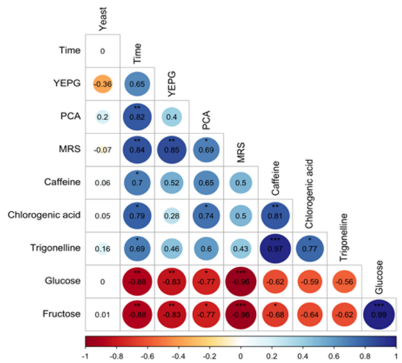

B

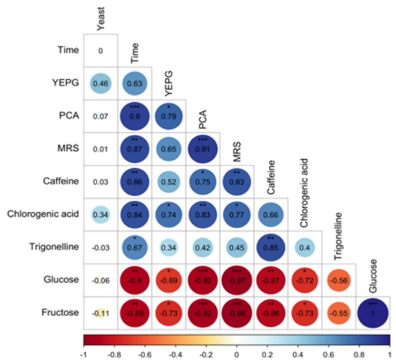

C

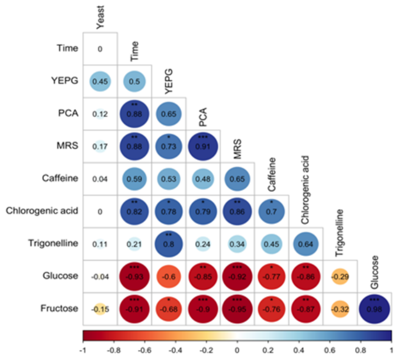

D

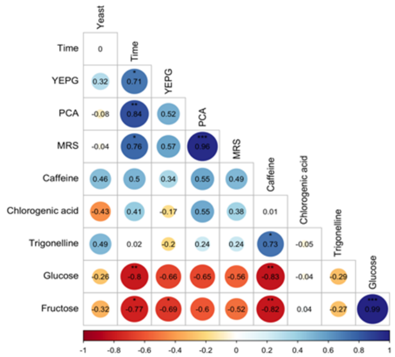

Supplement: Supplementary file 1 [file foods-14-00111-s001.zip › foods-3385201-supplementary.pdf]
